# Association of Female Reproductive Factors with Incident Cardiometabolic Disease: Finding from a European Population-Based Study

**DOI:** 10.5334/gh.1509

**Published:** 2025-12-26

**Authors:** Changxi Wang, Zhijie Lin, Fan Chen, Xiaoqian Zhu, Weize Lin, Ziqing Ruan, Jiabin Tu, Kaiyang Lin, Yansong Guo

**Affiliations:** 1 Department of Cardiology, Shengli Clinical Medical College of Fujian Medical University, Fujian Provincial Hospital, Fuzhou University Affiliated Provincial Hospital, Fuzhou, China; 2 Fujian Provincial Key Laboratory of Cardiovascular Disease, Fujian Provincial Center for Geriatrics, Fujian Provincial Clinical Research Center for Severe Acute Cardiovascular Diseases, Fuzhou, China; 3 Fujian Heart Failure Center Alliance, Fuzhou, China

**Keywords:** cardiometabolic disease, female reproductive factors, reproductive health, metabolic health

## Abstract

**Background:**

Cardiometabolic diseases (CMD), including ischemic heart disease, stroke, and type 2 diabetes, have caused an enormous global healthcare burden. Beyond traditional risk factors, female reproductive factors may also be associated with CMD. However, comprehensive evaluations of female reproductive factors related CMD is limited.

**Methods:**

A total of 189,411 women with no prior CMD from the UK Biobank cohort from 2007 to 2010 were included and followed until December 2022. Associations between reproductive factors and CMD were analyzed using Cox proportional hazards models with adjustment for potential confounders based on the directed acyclic graph (DAG).

**Results:**

During a median follow-up of 13.2 years, 17,251 incident CMD events occurred. Compared to menarche at age 12–13 years, <12 years and >13 years had a higher risk of CMD (HR <12 year (y) vs 12–13 y: 1.04 [95% CI, 1.01–1.08]; >13 y vs 12–13 y: 1.08 [1.04–1.13]). Earlier age at menopause was related to a higher risk of CMD (HR <46 y vs 50–51 y: 1.22 [1.15–1.29]; 46–49 y vs 50–51 y: 1.08 [1.03–1.14]), and a short reproductive lifespan (HR <33 y vs 36–38 y: 1.19 [1.13–1.25]; 33–35 y vs 36–38 y: 1.08 [1.03–1.14]). Younger age at first live birth (HR <22 y vs 24–26 y: 1.18 [1.12–1.24]; 22–23 y vs 24–26 y: 1.06 [1.00–1.12]) and last live birth (HR <26 y vs 29–30 y: 1.12 [1.06–1.18]) were associated with higher risk. Women with three or four children (HR 3–4 children: 1.21 [1.15–1.28]) and those with more than four children (HR >4 children: 1.27 [1.07–1.52]) were associated with higher risk of CMD. Recurrent pregnancy loss was associated with a 39% and 14% higher risk of CMD, respectively.

**Conclusion:**

Female reproductive factors are associated with CMD, independent of traditional risk factors. These reproductive factors could inform clinical screening and improve cardiometabolic risk assessment in women.

## Introduction

Cardiometabolic diseases (CMD), a cluster of conditions related to metabolic disorders and cardiovascular complications, including ischemic heart disease (IHD), stroke, and type 2 diabetes mellitus (T2DM), have caused an enormous global healthcare burden (1,2). Controlling the traditional risk factors, such as smoking, high blood pressure, body weight, and lipid profile, has greatly alleviated the disease burden (3). However, beyond traditional risk factors, an increasing number of studies have focused on female reproductive factors.

Recent studies have demonstrated that, although reproductive factors mostly occur in the early stages of life, they may have longer-term consequences. A review reported that early age at first live birth is associated with future cardiovascular disease (4). Studies from the China Kadoorie Biobank found a lifetime cumulative effect of reproductive factors on IHD and stroke (5,6). In addition, female reproductive factors shared several pathophysiological mechanisms with CMD, including insulin resistance (7), endothelial dysfunction (8), and inflammation (9). Some studies have suggested that reproductive factors such as age at menarche, age at menopause, multiparity, and miscarriages or stillbirths are associated with individual CMD types (10,11,12). However, few studies have simultaneously captured both metabolic and cardiovascular risk within composite CMD outcomes (13,14), and their results have been inconsistent. Hence, although numerous reproductive factors have been investigated in relation to individual CMD, the overall evidence linking reproductive factors and CMD remains limited.

Reproductive factors mostly occur early in life, while CMD often occurs later; various factors may affect their relationship, which in turn hinders the progress of related research and causes inconsistent results. Thus, it is essential to minimize potential confounders and to estimate the direct relationship between reproductive factors and CMD. Additionally, some guidelines have already incorporated age at menopause into risk assessment and stratification (15); however, current understanding of associations between other reproductive factors and CMD remains insufficient, particularly in elucidating the threshold at which these exposures shift from benign to harmful. Exploring the association and dose-response relationships between reproductive factors and CMD risk could improve understanding of CMD pathogenesis and facilitate early prevention strategies to modify potential risks.

Therefore, this study aimed to investigate systematically the relationship between female reproductive factors and CMD in a large, detailed population-based cohort, using a directed acyclic graph (DAG) to identify a minimally sufficient covariate set and reduce potential confounding.

## Method

### Data source

The UK Biobank is a large-scale prospective cohort study that recruited approximately 500,000 participants aged 37–73 years from 2007 to 2010 across 22 UK assessment centers. Baseline data were collected via self-reported questionnaires detailing socioeconomic status, lifestyle habits, reproductive factors, medical history, and other health-related variables. Anthropometric measurements and biological samples were collected using standardized procedures. All participants provided written informed consent. Ethical approval was granted by the North West Multi-Center Research Ethics Committee (protocols 11/NW/0382 and 16/NW/0274). This research was performed using the UK Biobank Resource (application No. 105322). Further details about the UK Biobank can be found on the showcase website (https://biobank.ndph.ox.ac.uk/showcase/).

### Study population

We included female participants in the UK Biobank (n = 272,532). And applied the following exclusion criteria for participants: 1. withdrawal of consent (n = 691); 2. with a history of CMD (including ischemic heart disease, stroke and diabetes) at baseline (n = 18,169); 3. missing information on reproductive factors (n = 25,773); 4. missing information on key covariates (n = 38,869). After exclusions, 189,411 participants were included for analysis (Figure S1). We then examined associations between reproductive factors and incident CMD in subsets with complete data. The link between menopausal age and new-onset CMD was assessed in postmenopausal women with recorded menopausal age (n = 112,203), whereas the relationships of age at first and last live birth with new-onset CMD were examined in parous women with two or more live births (n = 128,535).

### Assessment of women reproductive factors

Self-reported reproductive factors included in this study comprised age at menarche, age at menopause, reproductive lifespan (difference between age at menopause and age at menarche), age at first live birth (AFB), age at last live birth (ALB), number of live births, number of stillbirths, number of miscarriages or terminations, and history of hysterectomy or oophorectomy. Exogenous hormone uses encompassed menopausal hormone therapy and oral contraceptive use. Responses of ‘Prefer not to answer’ and ‘Do not know’ were treated as missing. All age-related variables were divided into tertiles or quintiles based on the population distribution. The number of live births were categorized into four groups (0, 1, 2, >2), while the numbers of stillbirths and of miscarriages or terminations were each categorized into three groups (0, 1, ≥2).

### Study outcome

The primary outcome in this study was incident CMD (a composite of ischemic heart disease, stroke, and type 2 diabetes). The secondary outcomes were the individual components: ischemic heart disease, stroke, and type 2 diabetes. These outcomes were identified using the International Classification of Diseases, 10th Revision (ICD-10) with codes I21–I25 for ischemic heart disease; I60–I64 for stroke; and E11 for type 2 diabetes (see Table S1 in the Supplement). Dates of incident CMD events were ascertained from four UK Biobank sources and defined as the earliest qualifying date across sources: 1. primary care data shared by various data suppliers and other intermediaries; 2. hospital admission dates retrieved via record linkage to Health Episode Statistics (England), the Patient Episode Database (Wales), and Scottish Morbidity Records; 3. death dates obtained from certificates held by the NHS Information Centre (England and Wales) and the NHS Central Register (Scotland); and 4. self-report at subsequent UK Biobank assessment center visit. Outcomes were censored at the date of first occurrence of the event, the date of death, or the administrative end of follow-up (set here to 31/12/2022), whichever came first.

### Covariates

We constructed a DAG (Figure S2) to identify the minimal adjustment set for associations between reproductive factors and CMD (16). This yielded the following covariates: age, Townsend Deprivation Index, ethnicity, education, smoking status, alcohol consumption, body mass index (BMI), inflammation level, lipid profile and insulin resistance. We used total cholesterol and high-density lipoprotein (HDL) as a representative of lipid profile; used triglyceride-glucose index (TyG) as a representative of insulin resistance (17) and used C-reactive protein (CRP) as a representative of inflammation level.

Socioeconomic status was assessed using the Townsend Deprivation Index, calculated from aggregated data on unemployment, car and house ownership, and household overcrowding linked to participants’ residential postcodes, with higher scores indicating greater levels of social deprivation. Self-reported data on ethnicity, smoking status, alcohol consumption, and medication use were collected via the touchscreen questionnaires during the initial visit to the assessment center. Responses of ‘Prefer not to answer’ and ‘Do not know’ were treated as missing. Ethnicity was classified as White or non-White. Education was classified based on whether participants had a college or university degree. Smoking status was defined as never, former, or current. Alcohol consumption was grouped into four categories: daily, 1–4 times/week, 1–3 times/month, and never. Triglycerides, cholesterol, high-density lipoprotein (HDL), glucose, and CRP were measured using the Beckman Coulter AU580. The TyG was calculated using the formula: ln [triglyceride (mg/dl) *blood glucose (mg/dl)/2] (17). Height, weight, and waist circumference were measured using standard procedures, and BMI was calculated as weight (kg) divided by the square of height (m). Overweight and obesity were defined as BMI >25 kg/m^2^ and >30 kg/m^2^, respectively (18). Abdominal obesity defined based on waist circumference, with a threshold of >80 cm for Asians and >88 cm for individuals of other ethnicities (19).

### Statistical analysis

Baseline characteristics were summarized as follows: continuous variables were reported as mean ± SD and compared using independent-sample t tests, while categorical variables were presented as frequencies (%) and compared using chi-square tests. Group differences were assessed using p-values and standardized mean differences (SMD).

Cox proportional hazards models were used to estimate hazard ratios (HRs) and 95% confidence intervals (CIs) for the association of female reproductive factors with new-onset CMD. Each reproductive factor was analyzed both continuously and categorically. Except for age of menarche, which was divided into tertiles, all other continuous variables were categorized into quintiles to form five groups for ease of interpretation; the middle group served as the reference. We further analyzed the relationship between continuous reproductive factors and incident CMD using restricted cubic splines (RCS) and performed likelihood ratio tests. The proportional hazards assumptions were tested using the Schoenfeld residuals and were not violated.

Two sequential models were adjusted for DAG-identified confounders. Model 1 was adjusted for age, ethnicity, education, Townsend index. Model 2 was further adjusted for BMI, smoking status, alcohol consumption, use of oral contraceptives, menopausal hormone therapy (HRT), HDL, CRP, and TyG. Predefined subgroup analyses were conducted by age group (categorized as > = 65 versus <65 years), smoking status (ever versus never smoker), BMI (normal, overweight, or obesity) and abdominal obesity (with or without), and the interaction between reproductive factors and subgroup factors were estimated using likelihood ratio test.

To verify the robustness of the results, we conducted several sensitivity analyses. First, given that reproductive lifespan may confound the association between age at menarche and CMD, we further evaluated this relationship after adjusting for reproductive lifespan. Second, we considered women who had only one child, treating ‘age of primiparous women at birth of child’ as ‘age at first live birth’ for more comprehensive data. Third, because the number of children could be a confounder, we added it to the adjusted model. Fourth, because surgically induced menopause and HRT may influence the association between reproductive lifespan and CMD. We incorporated the timing of hysterectomy and oophorectomy and the duration of HRT to derive a reproductive lifespan that reflects cumulative estrogen exposure. Fifth, we excluded CRP and TyG from the adjustment set to evaluate potential overadjustment. Sixth, we analyzed pregnancy loss using a more granular classification to capture differences in risk across types.

Two-sided P values were considered significant at P < 0.05. Statistical analyses were performed with the use of R version 4.4.1.

## Results

### Baseline characteristics

After applying the exclusion criteria, a total of 189,411 female participants were included in the primary analysis (Figure S1). During a median follow-up of 13.2 years, 17,251 cases of CMD were reported (Table 1). At baseline, the mean age of the participants was 55.88 ± 7.98 years, with 91.3% being of Whit. Compared to women who did not develop CMD, those who did had higher BMI (29.06 vs 26.55 kg/m^2^, SMD 0.472), larger waist circumference (90.01 vs 82.23 cm, SMD 0.540), higher systolic blood pressure (141.74 vs 133.82 mmHg, SMD 0.393), CRP level (3.86 vs 2.45 mg/L, SMD 0.296) and TYG (8.82 vs 8.55, SMD 0.523), poorer lipid profiles (Total cholesterol 6.02 vs 5.92 mmol/L, SMD: 0.090; HDL 1.49 vs 1.62 mmol/L, SMD 0.341), and a higher prevalence of current smoking (13.2% vs 8.3%, SMD 0.181). The average age at menarche was 12.97 ± 1.60, and 59.1% of participants were postmenopausal, with a mean menopausal age of 49.79 ± 5.00 years. Moreover, 67.9% of the women had at least two children, with an average AFB of 25.49 ± 4.61 years and ALB of 30.36 ± 4.88 years. On average, women had 1.8 children; 2.4% of women experience at least one stillbirth, and over 30% of women experience at least one miscarriage or termination (Table 1). Baseline characteristics according to grouping reproductive factors are shown in the supplementary materials (Table S2–S6).

**Table 1:** Baseline characteristics and reproductive factors of female by cardiometabolic disease status.


	TOTAL (n = 189,411)	WITHOUT CMD (n = 172,160)	WITH CMD (n = 17,251)	P	SMD

**Age, y**	55.88 (7.98)	55.49 (7.96)	59.71 (7.18)	<0.001	0.557

**TDI**	–1.47 (2.96)	–1.51 (2.93)	–1.01 (3.18)	<0.001	0.164

**Qualifications**					

College	32.7	33.7	22.5	<0.001	0.250

**Ethnicity**					

White	91.3	91.3	91.3	0.922	0.001

**BMI, kg/m^2^**	26.78 (4.96)	26.55 (4.81)	29.06 (5.78)	<0.001	0.472

**Waist circumference, cm**	83.84 (11.99)	82.23 (11.67)	90.01 (13.39)	<0.001	0.540

**Smoking status**				<0.001	0.181

Never	60.0	60.6	53.3		

Previous	31.3	31.1	33.5		

Current	8.7	8.3	13.2		

**Drinking status**				0.498	0.012

Never	19.7	19.8	19.5		

1–3 times per month	11.1	11.1	10.9		

1–4 times per week	48.8	48.7	49.2		

Daily	20.4	20.4	20.3		

**Age at menarche**	12.97 (1.60)	12.98 (1.60)	12.92 (1.68)	<0.001	0.041

**Group**				<0.001	0.074

<12	38.7	45.0	41.3		

12–13	44.6	38.4	41.4		

>13	16.7	16.6	17.3		

**Age at menopause**	49.79 (5.00)	49.84 (4.92)	49.38 (5.66)	<0.001	0.089

**Group**				<0.001	0.119

<46	20.6	20.1	24.2		

46–49	31.5	31.7	30.4		

50–51	19.7	20.0	17.3		

52–53	13.2	13.3	12.0		

>53	15.1	14.9	16.2		

**Reproductive lifespan**	36.82 (5.24)	36.87 (5.16)	36.44 (5.88)	<0.001	0.078

**Group**				<0.001	0.101

<33	21.1	20.8	24.4		

33–35	19.4	19.4	18.8		

36–38	28.4	28.7	25.7		

39–40	15.0	15.1	14.3		

>40	16.2	16.1	16.9		

**Age at first live birth**	25.49 (4.61)	25.64 (4.62)	24.08 (4.28)	<0.001	0.350

**Group**				<0.001	0.347

<22	27.3	26.1	38.7		

22–23	16.0	15.8	18.1		

24–26	24.9	25.7	22.7		

27–29	18.1	18.7	12.9		

>29	13.6	14.3	7.5		

**Age at last live birth**	30.36 (4.88)	30.46 (4.87)	29.46 (4.85)	<0.001	0.206

**Group**				<0.001	0.210

<26	22.3	21.6	29.0		

26–28	22.9	22.7	24.8		

29–30	15.7	15.8	14.4		

31–34	23.5	23.9	19.5		

>34	15.6	16.0	12.3		

**Number of live births**	1.80 (1.18)	1.79 (1.17)	1.99 (1.25)	<0.001	0.165

**Group**				<0.001	0.154

0	32.1	32.5	28.2		

1–2	62.0	61.9	62.7		

3–4	5.5	5.2	8.3		

>4	0.4	0.4	0.8		

**Number of stillbirths**				<0.001	0.062

0	97.7	97.8	96.7		

1	2.1	2.0	2.8		

> = 2	0.3	0.2	0.5		

**Number of miscarriages or terminations**				0.009	0.025

0	68.7	68.7	69.0		

1	20.8	20.9	20.0		

> = 2	10.5	10.5	11.0		

**Oral contraceptive pill**				<0.001	0.185

No	17.7	17.0	24.5		

Yes	82.3	83.0	75.5		

**HRT**				<0.001	0.311

No	63.6	65.0	49.8		

Yes	36.4	35.0	50.2		

**hysterectomy**				<0.001	0.250

No	82.8	83.8	73.6		

Yes	17.2	16.2	26.4		

**oophorectomy**				<0.001	0.147

No	92.2	92.6	88.2		

Yes	7.8	7.4	11.8		

**SBP, mmHg**	134.54 (19.73)	133.82 (19.47)	141.74 (20.81)	<0.001	0.393

**CRP, mg/L**	2.58 (4.20)	2.45 (4.04)	3.86 (5.39)	<0.001	0.296

**Triglycerides, mmol/L**	1.52 (0.83)	1.48 (0.80)	1.88 (1.01)	<0.001	0.441

**Total cholesterol, mmol/L**	5.93 (1.10)	5.92 (1.09)	6.02 (1.17)	<0.001	0.090

**LDL, mmol/L**	3.67 (0.85)	3.65 (0.84)	3.78 (0.91)	<0.001	0.147

**HDL, mmol/L**	1.61 (0.37)	1.62 (0.37)	1.49 (0.37)	<0.001	0.341

**Lp(a), nmol/L**	45.30 (49.38)	45.02 (49.16)	48.16 (51.26)	<0.001	0.063

**Oestradiol, pmol/L**	541.11 (472.49)	543.76 (472.30)	492.57 (473.39)	<0.001	0.108

**TYG**	8.57 (0.51)	8.55 (0.50)	8.82 (0.53)	<0.001	0.523

**Lipid-lowing drug**				<0.001	0.371

No	91.3	92.4	79.9		

Yes	8.7	7.6	20.1		

**Antihypertensive drug**				<0.001	0.444

No	85.5	87.1	69.2		

Yes	14.5	12.9	30.8		


Values are n (%) or mean (SD). Abbreviations: SMD, standardized mean difference; TDI, Townsend Deprivation Index; BMI, body mass index; SBP, systolic blood pressure; CRP, C-reactive protein; LDL, low-density lipoprotein; HDL, high-density lipoprotein; Lp(a), lipoprotein(a); TYG, triglyceride-glucose index. HRT, menopausal hormone therapy.

### Factors related to menarche and menopause

**Figure 1:**
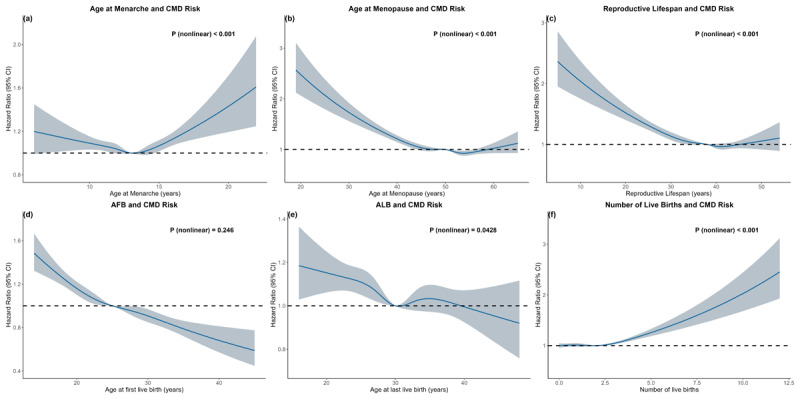
The RCS curve of the association between **(a)** age at menarche, **(b)** age at menopause, **(c)** reproductive lifespan, **(d)** age at first live birth, **(e)** age at last live birth, **(f)** number of live births and cardiometabolic disease in women in the UK Biobank. HR and 95% CI. Abbreviations: HR, hazard ratio; CI, confidence interval; CMD, cardiometabolic disease; AFB, age at first live birth; ALB, age at last live birth. Adjusted for age, Townsend deprived index, qualifications, ethnicity, smoking status, drinking status, body mass index, systolic blood pressure, C-reactive protein, cholesterol, high-density lipoprotein, triglyceride-glucose index, hormone replacement therapy and use of oral contraceptives.

In the fully adjusted model, nonlinear relationships with incident CMD were observed for age at menarche, age at menopause, and reproductive lifespan (Figure 1). After categorizing these variables based on their age distributions, both early and late menarche were significantly associated with an increased risk of CMD relative to the reference group (HR menarche <12 y: 1.04 [95% CI, 1.01–1.08]; >13 y: 1.08 [95% CI, 1.04–1.13]) (Table 2). Moreover, early menopause was linked to a higher risk of CMD (HR menopause <46 y: 1.22 [95% CI, 1.15–1.29]; 46–49 y: 1.08 [95% CI, 1.03–1.14]) compared to the reference group. Similarly, a shorter reproductive lifespan was associated with an elevated risk of CMD (HR reproductive lifespan <33 y: 1.19 [95% CI, 1.13–1.25]; 33–35 y: 1.08 [95% CI, 1.03–1.14]) relative to the reference group (Table 2). Secondary analyses of individual outcomes of IHD, stroke, and T2DM yielded similar results, except that early menarche was not associated with T2DM risk (Table S7–S9).

**Table 2:** Association of reproductive factors with incident cardiometabolic disease.


REPRODUCTIVEFACTORS	MODEL 1			MODEL 2		
	
	HR (95%CI)	P-VALUE	P FOR TREND	HR (95%CI)	P-VALUE	P FOR TREND

**Age at menarche, year**			<0.001			<0.001

<12	1.16 (1.12–1.20)	<0.001		1.04 (1.01–1.08)	0.011	

12–13	Ref.			Ref.		

>13	1.07 (1.02–1.11)	0.003		1.08 (1.04–1.13)	<0.001	

**Age at menopause^a^, year**			0.029			0.020

<46	1.33 (1.26–1.41)	<0.001		1.22 (1.15–1.29)	<0.001	

46–49	1.10 (1.04–1.16)	<0.001		1.08 (1.03–1.14)	0.004	

50–51	Ref.			Ref.		

52–53	1.02 (0.95–1.09)	0.595		1.01 (0.94–1.08)	0.856	

>53	1.07 (1.00–1.13)	0.044		1.02 (0.95–1.08)	0.627	

**Reproductive lifespan^a^, year**			0.388			0.036

<33	1.28 (1.22–1.35)	<0.001		1.19 (1.13–1.25)	<0.001	

33–35	1.08 (1.02–1.14)	0.005		1.08 (1.03–1.14)	0.004	

36–38	Ref.			Ref.		

39–40	1.03 (0.97–1.09)	0.350		1.01 (0.95–1.07)	0.696	

>40	1.05 (0.99–1.11)	0.085		0.98 (0.93–1.04)	0.591	

**Age at first live birth^b^, year**			<0.001			<0.001

<22	1.34 (1.28–1.41)	<0.001		1.18 (1.12–1.24)	<0.001	

22–23	1.11 (1.05–1.17)	<0.001		1.06 (1.00–1.12)	0.041	

24–26	Ref.			Ref.		

27–29	0.90 (0.84–0.95)	<0.001		0.94 (0.88–1.00)	0.053	

>29	0.78 (0.72–0.84)	<0.001		0.85 (0.79–0.92)	<0.001	

**Age at last live birth^b^, year**			<0.001			0.269

<26	1.20 (1.14–1.28)	<0.001		1.12 (1.06–1.18)	<0.001	

26–28	1.07 (1.01–1.13)	0.026		1.04 (0.98–1.11)	0.161	

29–30	Ref.			Ref.		

31–34	0.98 (0.92–1.05)	0.589		1.01 (0.95–1.07)	0.829	

>34	0.99 (0.93–1.06)	0.813		1.04 (0.97–1.11)	0.261	

**Number of live births**			<0.001			<0.001

0	1.01 (0.98–1.05)	0.554		1.01 (0.98–1.05)	0.460	

1–2	Ref.			Ref.		

3–4	1.32 (1.25–1.39)	<0.001		1.21 (1.15–1.28)	<0.001	

>4	1.48 (1.24–1.76)	<0.001		1.27 (1.07–1.52)	0.006	

**Number of stillbirths**			<0.001			0.006

0	Ref.			Ref.		

1	1.14 (1.04–1.25)	0.005		1.06 (0.97–1.16)	0.203	

> = 2	1.54 (1.24–1.91)	<0.001		1.39 (1.12–1.73)	0.003	

**Number of miscarriages or terminations**			<0.001			<0.001

0	Ref.			Ref.		

1	1.03 (0.99–1.07)	0.165		1.01 (0.98–1.05)	0.509	

> = 2	1.18 (1.12–1.24)	<0.001		1.14 (1.09–1.20)	<0.001	

**hysterectomy**						

No	Ref.			Ref.		

Yes	1.39 (1.34–1.44)	<0.001		1.20 (1.15–1.24)	<0.001	

**oophorectomy**						

No	Ref.			Ref.		

Yes	1.30 (1.24–1.36)	<0.001		1.10 (1.05–1.16)	<0.001	


Abbreviations: HR, hazard ratio; CI, confidence interval. Model 1 adjusted for age, Townsend deprived index, qualifications and ethnicity. Model2 adjusted for covariables in model 1 plus smoking status, drinking status, body mass index, systolic blood pressure, C-reactive protein, total cholesterol, high-density lipoprotein, triglyceride-glucose index, menopausal hormone therapy and use of oral contraceptives. ^a^Among postmenopausal women with valid menopausal age (n = 112,203). ^b^Among parous women (more than 2 children) with live birth age information (n = 128,535). Ref. = reference.

### Factors related to childbirth and pregnancy

ALB and the number of live births exhibited nonlinear associations with incident CMD, whereas AFB showed a linear relationship (Figure 1). Early AFB was associated with an increased risk of CMD (HR AFB <22 y: 1.18 [95% CI, 1.12–1.24]; 22–23 y: 1.06 [95% CI, 1.00–1.12]) relative to the reference group, while later AFB was linked to lower risks (HR AFB 27–29 y: 0.94 [95% CI, 0.88–1.00]; >29 y: 0.85 [95% CI, 0.79–0.92]). ALB was also associated with CMD risk, A younger age (<26 years) significantly linked to increased risk (HR ALB <26 y: 1.12 [95% CI, 1.06–1.18]), whereas later age was not significantly associated. Women with three or four children had an increased risk of CMD (HR: 1.21 [95% CI, 1.15–1.28]), and those with more than four children had the highest risk (HR: 1.27 [95% CI, 1.07–1.52]) compared to women with one or two children (Table 2). Furthermore, women with more than two stillbirths had an increased risk of CMD (HR 2≥ stillbirths: 1.39 [95% CI, 1.12–1.73]) compared to those without a history of stillbirth, while women with more than two miscarriages or terminations exhibited an elevated risk (HR ≥2 miscarriages/terminations: 1.14 [95% CI, 1.09–1.20]) relative to those without such a history (Table 2). Similar results were obtained in the secondary analysis of individual IHD, stroke, and T2DM, except that women with more than four children were no longer significantly correlated with IHD or stroke (Table S7–S9).

### Subgroup analysis

**Figure 2:**
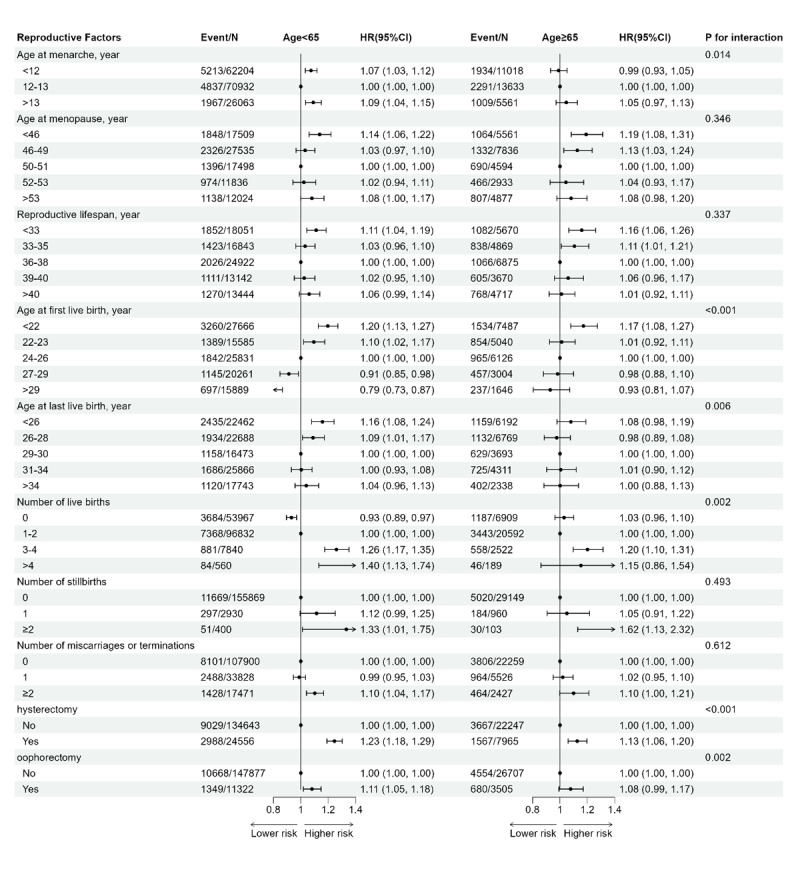
Forest plot of hazard ratios and 95% CIs for the association of reproductive factors and cardiometabolic disease stratified by age. Abbreviations: HR, hazard ratio; CI, confidence interval. Adjusted for Townsend deprived index, qualifications, ethnicity, smoking status, drinking status, body mass index, systolic blood pressure, C-reactive protein, cholesterol, high-density lipoprotein, triglyceride-glucose index, hormone replacement therapy and use of oral contraceptives.

Predefined subgroup analysis, by comparing participants 65 years or older with those younger than 65 years (Figure 2, Table S10), revealed a significant interaction (P for interaction = 0.014) between age at menarche and age subgroup, while both early and late age at menarche were associated with increased risk of CMD (HR menarche <12 y: 1.07 [95% CI, 1.03–1.12]; >13 y: 1.10 [95% CI, 1.04–1.15]) among women younger than 65 years, this relationship was not observed in those aged 65 or older (HR menarche <12 y: 0.99 [95% CI, 0.93–1.05]; >13 y: 1.05 [95% CI, 0.97–1.13]). Among the younger group, a later AFB was linked to a reduced risk of CMD (HR AFB >29 y: 0.79 [95% CI, 0.73–0.87]), but this association was not significant in the older group (HR AFB >29 y: 0.93 [95% CI, 0.81–1.07]). Additionally, a higher number of live births was related to an elevated risk of CMD in both groups, with a stronger effect observed in the younger group (HR 3–4 children: 1.26 [95% CI, 1.17–1.35] in younger vs 1.20 [95% CI, 1.10–1.31] in older; HR >4 children: 1.40 [95% CI, 1.13–1.74] in younger vs 1.15 [95% CI, 0.86–1.54] in older). All other associations of reproductive factors with CMD were largely consistent with the main findings.

**Figure 3:**
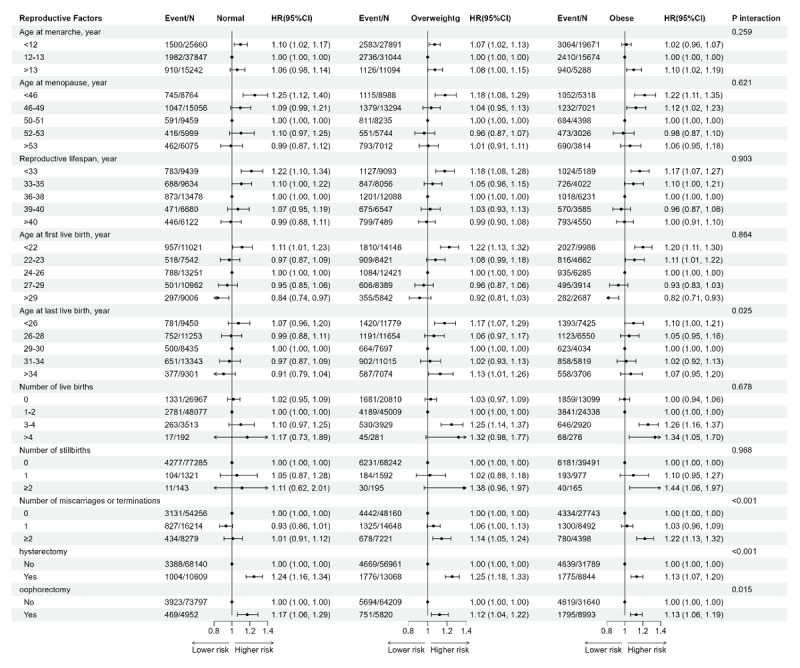
Forest plot of hazard ratios and 95% CIs for the association of reproductive factors and cardiometabolic disease stratified by BMI. Abbreviations: HR, hazard ratio; CI, confidence interval. Adjusted for age, Townsend deprived index, qualifications, ethnicity, smoking status, drinking status, systolic blood pressure, C-reactive protein, cholesterol, high-density lipoprotein, triglyceride-glucose index, hormone replacement therapy and use of oral contraceptives.

In the subgroup analysis based on BMI (Figure 3, Table S11), compared to overweight or obese women, the risk of CMD in women with a normal BMI was less influenced by reproductive factors. ALB, number of births, and pregnancy loss were no longer associated with an increased risk of CMD in the normal subgroup, whereas these associations persisted in the overweight and obese groups. Furthermore, we conducted subgroup analysis stratified by abdominal obesity, and pregnancy loss was similarly no longer significant in the normal subgroup (Figure S3, Table S12).

Among the smoking subgroup analysis, results similar to the main findings were observed, with no significant interactions observed (Figure S4, Table S13).

### Sensitivity analysis

Sensitivity analyses were conducted to assess the robustness of our findings. We observed a negative correlation between age at menarche and reproductive lifespan (Pearson correlation coefficient = –0.30, Figure S5). Therefore, reproductive lifespan was included in the multivariable model examining age at menarche and incident CMD; after this adjustment, late menarche (>13year) was no longer association with CMD (Table S14). Conversely, when age at menarche was added to the model for reproductive lifespan, the association remained unchanged (Table S15). Next, the number of live births was included in the model for reproductive factors, and the results were similar to the main analysis (Table S16).

When reproductive lifespan was recalculated using the definition that incorporated surgical menopause and HRT, the associations with CMD were essentially unchanged (Table S17), except that a substantially longer reproductive lifespan was associated with a higher risk of incident CMD.

We also considered women who had only one child, treating ‘age of primiparous women at birth of child’ as ‘age at first live birth’. We evaluated the relationship between the age at first live birth and CMD in the complete population (n = 153,690). The results of sensitivity analyses are similar to the main results (Table S18). In addition, when pregnancy loss was analyzed as separate categories, higher numbers of spontaneous miscarriages and pregnancy terminations were consistently associated with an increased risk of CMD (Table S19).

To address the potential risk of overadjustment, we repeated the analyses after excluding CRP and TyG from the adjustment set, and the results were not materially changed (Table S20).

## Discussion

In this prospective study of 189,411 female participants, we found that age at menarche, age at menopause, reproductive lifespan, ALB, and number of live births exhibited non-linear relationships with CMD. Specifically, both early and late age at menarche, early age at menopause, and a shorter reproductive lifespan were significantly associated with an increased risk of incident CMD. Early age at first live birth (AFB), early age at last live birth (ALB), and a higher number of live births, were correlated with an increased risk of CMD. A history of multiple (≥2) stillbirths, miscarriages, or terminations were linked to an elevated CMD risk. Women who had undergone hysterectomy or oophorectomy demonstrated a higher propensity of developing CMD.

### From menarche to menopause

Menarche is a milestone in a woman’s life as it denotes the start of reproductive capacity and is a central event in female puberty (20). In our study, a U-shaped association was found between age at menarche and risks of CMD. We found that both early and late age at menarche were associated with an increased incidence of CMD events. Consistently, previous research demonstrated that menarche age was associated with cardiometabolic disease (21,22,23). After further adjusting for reproductive lifespan, the association between late menarche and CMD was no longer significant, indicating that the increased CMD risk observed in women with late menarche may be attributable to their shortened reproductive lifespan. However, the observed association of early menarche remained significantly associated with CMD risk appear somewhat contradictory. Indeed, some studies have indicated that early menarche is linked to insulin resistance and metabolic risk (24,25), which could be the potential mechanism for the link between early menarche and CMD risk. In the subgroup analysis based on BMI and waist circumference, we found that in people with normal weight or waist circumference, earlier menarche was associated with increased risk of CMD, while in people with obese or abdominal obesity, only late menarche was associated with CMD risk. However, we did not observe significant interactions between menarche age and BMI, as well as waist circumference. Further research is still needed to study the causal relationship between menarche and obesity and how it jointly affects CMD risks.

Age at menopause is considered a marker or predictor of both reproductive and somatic aging, and of cardiometabolic and overall women’s health (26). We found that early menopause and shorter reproductive lifespan were associated with increased CMD risk. Endogenous estrogen exposure may be one of the causes. Prior studies have suggested that women with early menopause and consequently a shorter reproductive period with fewer cycles and lower cumulative exposure to endogenous sex hormones, have an increased risk of CMD (26,27,28). The beneficial effect of endogenous estrogen on the cardiovascular system has been well-established (29). As estrogen levels begin to decline after menopause, women become more predisposed to adverse cardiometabolic risk factors like insulin resistance, dyslipidemia, and endothelial dysfunction (8). Notably, we found that women who had undergone surgical menopause (hysterectomy or oophorectomy) also exhibited an elevated risk of CMD. This could be a confounder for the association between menopause and CMD; however, after excluding these women, the association remained significant. This indicated that both natural and surgical menopause are related to an elevated risk of CMD. This finding is consistent with previous studies (30,31). Previous guidelines recommend age at menopause <40 years are a risk factor for cardiovascular disease (15). In our study, menopause before 46 years was associated with a 22% higher CMD risk. Therefore, future research needs to focus on which age threshold is appropriate for risk stratification.

In the subgroup analysis stratified by age, we observed a significant interaction between the age group and age at menarche. The relationship between age at menarche and CMD was no longer significant in older adults, suggesting that advanced age may offset the impact of age at menarche on CMD. This interesting finding may be attributed to the different CMD risks of each group. Old age itself is a potent risk factor for CMD risk, and the old age group is more likely to have other traditional risk factors, which results in the null or smaller effect size of reproductive factors in old-aged women.

### Age at first and last live birth and parity

Current studies suggest that younger AFB may be associated with increased IHD, stroke and T2DM (4,10), as well as with adverse cardiometabolic health, such as adiposity, adverse lipid profiles, and insulin resistance (32,33). Our study confirmed that younger AFB was a risk factor for incident CMD. Similarly, an earlier age at last birth (ALB) was associated with higher CMD risk. This finding may reflect the restriction to women with ≥2 live births, among whom those with earlier ALB also tended to have earlier AFB. In the sensitivity analysis that included women with one child, the relationship between AFB and CMD remained significant. Two potential mechanisms may explain these associations. First, women with younger age at first live birth are more likely to have a larger number of parities, and multiparity has been proved to be a risk factor for CMD in our study. Similarly, previous research also supports this notion (13). Pregnancy often induces fluctuations in cardiometabolic risk factors. For example, blood lipids and blood pressures go up and down during pregnancy (34,35). Weight gain during pregnancy and redistribution after pregnancy (36), and insulin exhibit an upward trend due to the effects of gestational hormones during pregnancy (37). Although most changes are reversible, the long-term fluctuations in hormonal and cardiometabolic risk factors associated with multiparity may induce lasting alterations and increase CMD risk (38,39,40). However, adjustment for the number of live births in our sensitivity analyses did not change the results, indicating that multiparity cannot fully account for the observed differences. Second, women with younger AFB are more likely to have lower education level, lower socioeconomic status and poor health status (41,42,43). Previous research demonstrated that social, cultural, and behavioral factors may have a larger contribution to the association of younger AFB with worse cardiometabolic risk profiles (32). Our study adjusted for Townsend Deprivation Index, education, and ethnicity, yet the results remained significant. Future research should aim to identify the specific social patterns that affect reproductive age and cardiometabolic disease by comprehensively considering individuals’ socioeconomic status, which could help identify high-risk groups and provide targeted support or interventions.

Subgroup analyses revealed significant interactions between age group and AFB, ALB, and parity. The effects of these reproductive factors on CMD risk were attenuated in the older group. Among women with three to four children, an increased risk was observed regardless of age. However, in the older subgroup with more than four children, a counterintuitive lack of significant risk elevation was noted, possibly due to the limited number of individuals in that subgroup.

### Pregnancy loss

Previous studies have reported that women with a history of pregnancy loss have an increased risk of coronary heart disease (44), stroke (11), and diabetes (12). However, some studies have yielded inconsistent results (45). Some studies have assessed the relationship between pregnancy loss and composite CMD outcome (13,46), yielding similar findings. However, these studies are limited by their cross-sectional design, which may not fully capture causality. In our study, after adjusting for confounders, we found that recurrent pregnancy loss, whether it was stillbirth, miscarriage, or terminations, was associated with an increased risk of CMD.

Pregnancy loss may affect short- and long-term cardiometabolic trajectories, inducing CMD in women later in life (47,48). There are several possible explanations for the mechanisms underlying the link between pregnancy loss and cardiometabolic disease. First, studies have demonstrated that women with recurrent pregnancy loss have a higher rate of endothelial dysfunction compared to those with uncomplicated pregnancies (49); endothelial dysfunction is one of the causes of CMD. Second, pregnancy loss is an autoimmune-like process (50,51) that can trigger systemic inflammation (9). Systemic inflammation is closely linked to CMD (52,53), suggesting that inflammation may play a crucial role in both miscarriage and CMD. Third, pregnancy loss and CMD share some risk factors and pathophysiological mechanisms. For example, metabolic risk factors, such as dyslipidemia and obesity, are associated with both pregnancy loss and CMD risk (54,55,56). Unhealthy behaviors, such as smoking and drinking are also associated with both pregnancy loss and CMD risk (57,58). After adjusting for the relevant risk factors, the results remained statistically significant, suggesting that unidentified potential factors may exist. Future research should explore the other underlying shared risk factors and elucidate their pathogenic mechanisms to provide a basis for precise prevention and treatment.

In subgroup analysis by BMI and abdominal obesity, we observed significant interactions between obesity and pregnancy loss. In subgroups with normal body weight or shape, miscarriage or terminations were not related to an increased risk of CMD; however, these associations were observed in those with obesity or abdominal obesity, which suggests that controlling weight and body shape may help avoid the adverse cardiometabolic risks associated with miscarriage and abortion. Further research is needed to establish a causal relationship between obesity and miscarriage or terminations.

### Study strengths and limitations

This study has several advantages. First, we present findings from a large population-based study of 189,411 women with a long follow-up, and detailed information on reproductive factors. Second, using a DAG-guided, minimally sufficient adjustment set, we showed that female reproductive factors are independent risk factors for cardiometabolic disease beyond traditional risk factors. There are a few limitations. First, although we used different criteria across ethnic groups, most participants were White, limiting the further expansion of the results to the entire population, particularly among South Asians. Prior studies indicate that reproductive profiles in South Asian women may differ from those in European populations, and the corresponding associations may therefore vary in this group. Second, information on reproductive factors was collected via self-reported questionnaires, which may lead to recall bias. Third, a healthy volunteer selection bias of UK Biobank has been previously reported 40 which may underestimate the true associations. Fourth, although we adjusted for traditional cardiovascular risk factors, they were single measurements at the baseline of the study, and we cannot rule out the possibility of confounding resulting from the changes of these confounders over time. Fourth, although we adjusted for traditional cardiovascular risk factors, they were single measurements at the baseline of the study, and we cannot rule out the possibility of confounding resulting from the changes of these confounders over time. The conclusions of this study still need to be confirmed by larger studies in other countries and different ethnic groups.

## Conclusions

Female reproductive factors are associated with CMD independent of traditional cardiometabolic risk factors. Our findings elucidate the threshold at which these reproductive factors shift from benign to harmful, thereby providing more accurate risk stratification for the identification and prevention of cardiometabolic disease.

## Data Accessibility Statement

The aggregated data supporting this article are available in the article and in the Supplementary Material.

## Additional File

The additional file for this article can be found as follows:

10.5334/gh.1509.s1 Supplementary Material. Tables S1–S20 and Figures S1–S5.

## Abbreviations

**CMD** cardiometabolic diseases

**DAG** directed acyclic graph

**IHD** ischemic heart disease

**T2DM** type 2 diabetes mellitus

**AFB** age at first live birth

**ALB** age at last live birth

**HRT** menopausal hormone therapy

**CRP** C-reactive protein

**HDL** high-density lipoprotein

**TyG** triglyceride-glucose index

**BMI** body mass index

**SMD** standardized mean differences

**RCS** restricted cubic splines

**HR** hazard ratio

**CI** confidence interval
