# Supplementary material for: Association of Female Reproductive Factors with Incident Cardiometabolic Disease: Finding from a European Population-Based Study
Source: Glob Heart. 2025 Dec 26;20(1):114. doi: 10.5334/gh.1509 (PMC12742377; doi:10.5334/gh.1509)
Supplement: Supplementary Material. — Tables S1–S20 and Figures S1–S5. [file gh-20-1-1509-s1.pdf]

Association of Female Reproductive Factors with Incident Cardiometabolic Disease: Finding from A European Population-Based Study

Supplementary Materials

Table S1. Definition of Cardiometabolic Diseases by ICD10 Code.

Table S2. Baseline Characteristics of Female by Different Group of Menarche Age.

Table S3. Baseline Characteristics of Female by Different Group of Menopause Age.

Table S4. Baseline Characteristics of Female by Different Group of Reproductive Lifespan.

Table S5. Baseline Characteristics of Female by Different Group of Age at First Live Birth.

Table S6. Baseline Characteristics of Female by Different Group of Age at Last Live Birth.

Table S7. Association of Reproductive Factors with Incident Ischemic Heart Disease.

Table S8. Association of Reproductive Factors with Incident Stroke.

Table S9. Association of Reproductive Factors with Incident Type 2 Diabetes Mellitus.

Table S10. Association of Reproductive Factors with Incident Cardiometabolic Disease Stratified by Age.

Table S11. Association of Reproductive Factors with Incident Cardiometabolic Disease Stratified by BMI.

Table S12. Association of Reproductive Factors with Incident Cardiometabolic Disease Stratified by Waist Circumference.

Table S13. Association of Reproductive Factors with Incident Cardiometabolic Disease Stratified by Smoking Status.

Table S14. Association of Menarche Age with Incident Cardiometabolic Disease Further adjusted by Reproductive Lifespan.

Table S15. Association of Reproductive Lifespan with Incident Cardiometabolic Disease Further adjusted by Menarche Age.

Table S16. Association of Reproductive Factors with Incident Cardiometabolic Disease Further adjusted Live Births.

Table S17. Associations between reproductive lifespan and incident cardiometabolic disease using menopause age redefined to include surgical menopause and HRT.

Table S18. Association of Age at First Live Birth with Incident Cardiometabolic Disease When Considering All Parous Women.

Table S19. Associations of distinct pregnancy-loss categories with incident cardiometabolic disease.

Table S20. Association of Reproductive Factors with Incident Cardiometabolic Disease after excluding CRP and TyG from the adjustment set.

Figure S1. Flowchart of the study population.

Figure S2. Causal directed acyclic graph of the association between reproductive factors and cardiometabolic disease.

Figure S3. Forest plot of hazard ratios and 95% CIs for the association of reproductive factors and cardiometabolic disease stratified by waist

circumference.

Figure S4. Forest plot of hazard ratios and 95% CIs for the association of reproductive factors and cardiometabolic disease stratified by smoking status.

Figure S5. Correlation Heatmap of Reproductive Factors.

Table S1. Definition of Cardiometabolic Diseases by ICD10 Code.

Disease	ICD-10 code
Ischemic Heart Disease	I21.0, I21.1, I21.2, I21.3, I21.4, I21.9, I22.0, I22.1, I22.8, I22.9, I24.0, I24.1, I24.8, I24.9, I25.0, I25.1, I25.2, I25.3, I25.4, I25.5, I25.6, I25.8, I25.9
Stroke	I60.0, I60.1, I60.2, I60.3, I60.4, I60.5, I60.6, I61.0, I61.1, I61.2, I61.3, I61.4, I61.5, I61.6, I61.9, I62.0, I62.1, I62.9, I63.0, I63.1, I63.2, I63.3, I63.4, I63.5, I63.6, I63.8, I63.9, I64.0
Type 2 Diabetes Mellitus	E11.0, E11.1, E11.2, E11.3, E11.4, E11.5, E11.6, E11.7, E11.8, E11.9

Abbreviations: ICD10, International Classification of Diseases, 10th Revision

Table S2. Baseline Characteristics of Female by Different Group of Menarche Age

	**<12 years (n=73222)**	**12-13 years (n=84565)**	**>13 years (n=31624)**	**P**	**SMD**
**Age, y**	56.02 (7.84)	55.77 (8.05)	55.84 (8.10)	<0.001	0.021
**TDI**	-1.50 (2.93)	-1.52 (2.93)	-1.23 (3.09)	<0.001	0.064
**Qualifications**					
College	33.5	33.9	27.5	<0.001	0.092
**Ethnic**					
White	91.7	91.2	90.6	<0.001	0.026
**BMI, kg/m^2**	27.61 (5.27)	26.36 (4.70)	25.99 (4.62)	<0.001	0.219
**Waist circumference, cm**	85.22 (12.53)	83.09 (11.60)	82.68 (11.44)	<0.001	0.141
**Smoking status**				<0.001	0.061
Never	60.8	60.1	57.5		
Previous	30.8	31.5	32		
Current	8.4	8.3	10.5		
**Drinking status**				0.329	0.012
Never	19.5	19.8	20.1		
1–3 times per month	11.2	11.1	11		
1–4 times per week	48.8	48.7	48.9		
Daily	20.5	20.4	20.1		
**SBP, mmHg**	135.42 (19.92)	134.07 (19.57)	133.74 (19.62)	<0.001	0.057
**CRP, mg/L**	2.75 (4.37)	2.46 (4.06)	2.52 (4.15)	<0.001	0.045
**Triglycerides, mmol/L**	1.56 (0.84)	1.49 (0.82)	1.50 (0.83)	<0.001	0.052
**Cholesterol, mmol/L**	5.94 (1.10)	5.92 (1.09)	5.91 (1.10)	<0.001	0.018
**LDL, mmol/L**	3.68 (0.85)	3.66 (0.85)	3.65 (0.85)	<0.001	0.023
**HDL, mmol/L**	1.60 (0.37)	1.62 (0.37)	1.61 (0.38)	<0.001	0.043

**Lp(a), nmol/L**	45.66 (49.57)	45.04 (49.33)	45.16 (49.08)	0.073	0.008
**Oestradiol, pmol/L**	537.91 (472.84)	547.85 (474.46)	530.09 (466.18)	0.01	0.025
**TYG**	8.60 (0.51)	8.55 (0.51)	8.55 (0.52)	<0.001	0.06
**Lipid-lowing drug**				<0.001	0.031
No	90.6	91.9	91.3		
Yes	9.4	8.1	8.7		
**Antihypertensive drug**				<0.001	0.051
No	83.8	86.5	86.5		
Yes	16.2	13.5	13.5		

Values are n (%) or mean SD.

Abbreviations: SMD, standardized mean difference; TDI, Townsend Deprivation Index; BMI, body mass index; SBP, systolic blood pressure; CRP, C-reactive protein; LDL, low-density lipoprotein; HDL, high-density lipoprotein; Lp(a), lipoprotein(a); TYG, triglyceride-glucose index. HRT, hormone replacement therapy.

Table S3. Baseline Characteristics of Female by Different Group of Menopause Age.

	**<46 years (n=23070)**	**46-49 years (n=35371)**	**50-51 years (n=22092)**	**52-53 years (n=14769)**	**>53 years (n=16901)**	**P**	**SMD**
**Age, y**	59.07 (6.69)	59.46 (5.66)	59.86 (4.95)	60.29 (4.37)	62.25 (3.62)	<0.001	0.296
**TDI**	-1.28 (3.07)	-1.53 (2.94)	-1.70 (2.82)	-1.76 (2.76)	-1.77 (2.77)	<0.001	0.083
**Qualifications**							
College	25.1	30.2	33.1	33	30.1	<0.001	0.084
**Ethnic**							
White	91.8	91.5	92.5	92.7	92.8	<0.001	0.025
**BMI, kg/m^2**	27.06 (4.96)	26.58 (4.75)	26.57 (4.76)	26.75 (4.71)	27.19 (4.78)	<0.001	0.071
**Waist circumference, cm**	84.81 (12.01)	83.76 (11.66)	83.66 (11.69)	84.06 (11.66)	85.41 (11.76)	<0.001	0.077
**Smoking status**						<0.001	0.126
Never	53.4	57.9	60.2	61.5	60.4		
Previous	35.1	33.5	33.4	32.5	34.5		
Current	11.5	8.6	6.4	5.9	5.1		
**Drinking status**						0.169	0.02
Never	20.1	19.7	19.7	19.5	19.2		
1–3 times per month	11	11.1	11.2	10.7	11.2		
1–4 times per week	48.1	48.9	48.6	49.4	48.5		
Daily	20.8	20.2	20.6	20.4	21.1		
**SBP, mmHg**	136.92 (19.93)	137.06(19.97)	137.96(19.75)	138.56(19.53)	140.90 (19.55)	<0.001	0.096
**CRP, mg/L**	2.92 (4.42)	2.57 (4.26)	2.50 (4.16)	2.53 (4.37)	2.66 (4.25)	<0.001	0.045

**Triglycerides, mmol/L**	1.64 (0.88)	1.57 (0.82)	1.56 (0.80)	1.57 (0.81)	1.64 (0.84)	<0.001	0.059
**Cholesterol, mmol/L**	6.08 (1.10)	6.14 (1.08)	6.15 (1.07)	6.17 (1.09)	6.18 (1.08)	<0.001	0.043
**LDL, mmol/L**	3.77 (0.86)	3.81 (0.84)	3.81 (0.84)	3.83 (0.84)	3.84 (0.85)	<0.001	0.037
**HDL, mmol/L**	1.61 (0.38)	1.65 (0.38)	1.65 (0.38)	1.65 (0.38)	1.63 (0.37)	<0.001	0.054
**Lp(a), nmol/L**	46.56 (50.20)	46.00 (49.61)	45.41 (49.47)	46.49 (50.28)	45.73 (49.71)	0.179	0.012
**Oestradiol, pmol/L**	405.41 (365.84)	382.54(318.60)	365.02 (269.72)	364.80(271.89)	352.75(288.23)	<0.001	0.078
**TYG**	8.66 (0.50)	8.62 (0.49)	8.62 (0.49)	8.63 (0.49)	8.68 (0.49)	<0.001	0.067
**Lipid-lowing drug**						<0.001	0.051
No	87.2	89.4	89.9	89.6	87.1		
Yes	12.8	10.6	10.1	10.4	12.9		
**Antihypertensive drug**						<0.001	0.057
No	82	83.5	83.3	82.4	78.6		
Yes	18	16.5	16.7	17.6	21.4		

Values are n (%) or mean SD.

Abbreviations: SMD, standardized mean difference; TDI, Townsend Deprivation Index; BMI, body mass index; SBP, systolic blood pressure; CRP, C-reactive protein; LDL, low-density lipoprotein; HDL, high-density lipoprotein; Lp(a), lipoprotein(a); TYG, triglyceride-glucose index. HRT, hormone replacement therapy.

Table S4. Baseline Characteristics of Female by Different Group of Reproductive Lifespan.

	<33 years (n=23721)	33-35 years (n=21712)	36-38 years (n=31797)	39-40 years (n=16812)	>40 years (n=18161)	P	SMD
**Age, y**	58.99 (6.67)	59.31 (5.81)	59.89 (5.11)	60.53 (4.52)	61.77 (3.86)	<0.001	0.266
**TDI**	-1.25 (3.09)	-1.49 (2.96)	-1.67 (2.83)	-1.75 (2.80)	-1.79 (2.74)	<0.001	0.092
**Qualifications**						<0.001	0.082
College	24.8	29.4	32.2	32.7	31.7		
**Ethnic**						<0.001	0.032
White	91.4	91.4	92.2	92.8	92.9		
**BMI, kg/m^2**	26.85 (4.88)	26.38 (4.65)	26.57 (4.73)	26.89 (4.79)	27.50 (4.92)	<0.001	0.107
**Waist circumference, cm**	84.45 (11.88)	83.46 (11.50)	83.70 (11.63)	84.40 (11.78)	85.70 (12.02)	<0.001	0.089
**Smoking status**						<0.001	0.132
Never	53.4	57.2	59.1	61.8	61.1		
Previous	34.9	33.9	33.7	32.5	33.9		
Current	11.7	8.9	7.2	5.7	5.1		
**Drinking status**						0.586	0.015
Never	20.1	20	19.6	19.5	19.4		
1–3 times per month	10.8	11	11.2	11.2	11		
1–4 times per week	48.3	48.7	48.7	49	48.7		
Daily	20.7	20.3	20.5	20.4	21		
**SBP, mmHg**	136.66 (19.93)	136.88 (20.05)	137.66 (19.76)	139.08 (19.40)	140.58 (19.75)	<0.001	0.101
**CRP, mg/L**	2.87 (4.38)	2.55 (4.26)	2.53 (4.21)	2.54 (4.31)	2.70 (4.31)	<0.001	0.04
**Triglycerides, mmol/L**	1.63 (0.88)	1.56 (0.82)	1.56 (0.80)	1.58 (0.83)	1.64 (0.83)	<0.001	0.054

**Cholesterol, mmol/L**	6.08 (1.10)	6.14 (1.08)	6.15 (1.07)	6.15 (1.08)	6.18 (1.08)	<0.001	0.039
**LDL, mmol/L**	3.78 (0.86)	3.81 (0.84)	3.82 (0.84)	3.81 (0.84)	3.84 (0.85)	<0.001	0.034
**HDL, mmol/L**	1.61 (0.38)	1.65 (0.38)	1.65 (0.38)	1.65 (0.38)	1.63 (0.37)	<0.001	0.052
**Lp(a), nmol/L**	46.58 (50.13)	45.30 (49.14)	46.10 (49.89)	46.07 (50.11)	45.97 (49.76)	0.185	0.011
**Oestradiol, pmol/L**	406.78 (368.79)	385.60(321.18)	372.85 (292.05)	351.86(254.50)	355.26 (278.03)	<0.001	0.09
**TYG**	8.65 (0.51)	8.62 (0.49)	8.62 (0.49)	8.64 (0.49)	8.68 (0.49)	<0.001	0.061
**Lipid-lowing drug**						<0.001	0.042
No	87.4	89.7	89.6	88.8	87.6		
Yes	12.6	10.3	10.4	11.2	12.4		
**Antihypertensive drug**						<0.001	0.065
No	82.4	84.1	83.4	81.6	78.7		
Yes	17.6	15.9	16.6	18.4	21.3		

Values are n (%) or mean SD.

Abbreviations: SMD, standardized mean difference; TDI, Townsend Deprivation Index; BMI, body mass index; SBP, systolic blood pressure; CRP, C-reactive protein; LDL, low-density lipoprotein; HDL, high-density lipoprotein; Lp(a), lipoprotein(a); TYG, triglyceride-glucose index. HRT, hormone replacement therapy.

Table S5. Baseline Characteristics of Female by Different Group of Age at First Live Birth.

	**<22 years (n=35153)**	**22-23 years (n=20625)**	**24-26 years (n=31957)**	**27-29 years (n=23265)**	**>29 years (n=17535)**	**P**	**SMD**
**Age, y**	58.09 (7.43)	58.28 (7.62)	57.26 (7.54)	55.46 (7.63)	53.29 (7.90)	<0.001	0.329
**TDI**	-0.74 (3.21)	-1.76 (2.78)	-2.17 (2.55)	-2.22 (2.50)	-1.95 (2.66)	<0.001	0.237
**Qualifications**						<0.001	0.48
College	12.9	19.4	30.7	43.7	53.4		
**Ethnic**						<0.001	0.081
White	92.9	93.6	93	91.6	88.7		
**BMI, kg/m^2**	27.90 (5.08)	27.12 (4.72)	26.62 (4.60)	26.10 (4.54)	25.72 (4.52)	<0.001	0.228
**Waist circumference, cm**	86.38 (12.16)	84.67 (11.52)	83.64 (11.40)	82.51 (11.26)	81.93 (11.23)	<0.001	0.191
**Smoking status**						<0.001	0.166
Never	51.4	61.7	64.9	65.5	62.5		
Previous	35.3	31	29.1	29.3	32.3		
Current	13.4	7.3	6	5.2	5.2		
**Drinking status**						0.634	0.014
Never	19.9	19.8	19.6	19.7	19.2		
1–3 times per month	10.9	10.9	11.3	11.3	11.1		
1–4 times per week	49	48.6	48.7	48.7	49.2		
Daily	20.3	20.8	20.4	20.3	20.5		
**SBP, mmHg**	137.25 (19.82)	137.68 (19.92)	136.05 (19.64)	133.60 (19.41)	130.19 (18.92)	<0.001	0.192
**CRP, mg/L**	3.13 (4.63)	2.71 (4.22)	2.50 (4.11)	2.22 (3.70)	2.10 (3.86)	<0.001	0.122
**Triglycerides, mmol/L**	1.68 (0.91)	1.60 (0.86)	1.53 (0.81)	1.44 (0.78)	1.35 (0.72)	<0.001	0.202

**Cholesterol, mmol/L**	6.00 (1.12)	6.02 (1.10)	5.99 (1.09)	5.91 (1.08)	5.78 (1.06)	<0.001	0.103
**LDL, mmol/L**	3.74 (0.87)	3.74 (0.85)	3.71 (0.85)	3.64 (0.84)	3.54 (0.82)	<0.001	0.117
**HDL, mmol/L**	1.55 (0.36)	1.59 (0.36)	1.62 (0.37)	1.64 (0.37)	1.64 (0.37)	<0.001	0.118
**Lp(a), nmol/L**	46.06 (49.73)	45.71 (49.55)	45.45 (49.77)	45.28 (49.47)	44.13 (48.74)	0.005	0.017
**Oestradiol, pmol/L**	523.65 (490.57)	532.59(429.23)	542.55 (449.41)	551.44(470.88)	563.49 (506.91)	<0.001	0.042
**TYG**	8.68 (0.51)	8.64 (0.51)	8.58 (0.50)	8.52 (0.51)	8.45 (0.50)	<0.001	0.228
**Lipid-lowing drug**						<0.001	0.149
No	87.3	88.4	91.3	93.3	95.2		
Yes	12.7	11.6	8.7	6.7	4.8		
**Antihypertensive drug**						<0.001	0.16
No	80.3	81.9	84.7	88.1	91.1		
Yes	19.7	18.1	15.3	11.9	8.9		

Values are n (%) or mean SD.

Abbreviations: SMD, standardized mean difference; TDI, Townsend Deprivation Index; BMI, body mass index; SBP, systolic blood pressure; CRP, C-reactive protein; LDL, low-density lipoprotein; HDL, high-density lipoprotein; Lp(a), lipoprotein(a); TYG, triglyceride-glucose index. HRT, hormone replacement therapy.

Table S6. Baseline Characteristics of Female by Different Group of Age at Last Live Birth.

	<26 years (n=28654)	26-28 years (n=29457)	29-30 years (n=20166)	31-34 years (n=30177)	>34 years (n=20081)	P	SMD
**Age, y**	58.47 (7.18)	58.05 (7.53)	56.85 (7.71)	55.56 (7.81)	54.30 (7.99)	<0.001	0.282
**TDI**	-1.24 (3.03)	-1.85 (2.75)	-1.96 (2.73)	-1.90 (2.74)	-1.51 (2.94)	<0.001	0.128
**Qualifications**						<0.001	0.355
College	14.1	22.8	31.7	38.7	45.1		
**Ethnic**						<0.001	0.138
White	94.7	94.2	93.2	90.9	86.7		
**BMI, kg/m^2**	27.60 (4.91)	27.01 (4.73)	26.65 (4.72)	26.44 (4.71)	26.25 (4.79)	<0.001	0.137
**Waist circumference, cm**	85.59 (11.86)	84.36 (11.59)	83.66 (11.55)	83.36 (11.55)	83.24 (11.76)	<0.001	0.097
**Smoking status**						<0.001	0.1
Never	54.2	61.7	63.7	62.9	60.7		
Previous	34.8	30.6	29.4	30.3	32.4		
Current	11	7.7	6.9	6.8	6.8		
**Drinking status**						0.771	0.012
Never	19.7	19.6	19.8	19.9	19.3		
1–3 times per month	11.3	11.1	11	11.1	11		
1–4 times per week	48.7	49	48.6	48.5	49.5		
Daily	20.3	20.4	20.6	20.6	20.3		
**SBP, mmHg**	137.81 (19.56)	137.35 (19.92)	135.47 (19.63)	133.77 (19.56)	131.46 (19.36)	<0.001	0.166
**CRP, mg/L**	2.98 (4.52)	2.71 (4.27)	2.52 (4.11)	2.40 (4.03)	2.26 (3.83)	<0.001	0.083
**Triglycerides, mmol/L**	1.67 (0.89)	1.58 (0.84)	1.53 (0.83)	1.47 (0.80)	1.42 (0.78)	<0.001	0.143

**Cholesterol, mmol/L**	6.04 (1.10)	6.01 (1.10)	5.97 (1.08)	5.90 (1.09)	5.80 (1.08)	<0.001	0.107
**LDL, mmol/L**	3.76 (0.86)	3.73 (0.86)	3.70 (0.84)	3.65 (0.84)	3.58 (0.83)	<0.001	0.108
**HDL, mmol/L**	1.58 (0.37)	1.60 (0.37)	1.61 (0.37)	1.62 (0.37)	1.61 (0.37)	<0.001	0.047
**Lp(a), nmol/L**	46.01 (49.76)	45.03 (49.50)	45.35 (49.66)	45.59 (49.46)	45.13 (49.22)	0.228	0.01
**Oestradiol, pmol/L**	528.37 (451.95)	535.86(449.37)	531.14 (442.50)	549.46(461.89)	562.39 (541.59)	0.001	0.036
**TYG**	8.67 (0.51)	8.62 (0.50)	8.58 (0.51)	8.54 (0.51)	8.50 (0.51)	<0.001	0.169
**Lipid-lowing drug**						<0.001	0.111
No	87.6	89.2	91.1	92.5	93.9		
Yes	12.4	10.8	8.9	7.5	6.1		
**Antihypertensive drug**						<0.001	0.112
No	80.9	82.6	84.7	86.9	88.8		
Yes	19.1	17.4	15.3	13.1	11.2		

Values are n (%) or mean SD.

Abbreviations: SMD, standardized mean difference; TDI, Townsend Deprivation Index; BMI, body mass index; SBP, systolic blood pressure; CRP, C-reactive protein; LDL, low-density lipoprotein; HDL, high-density lipoprotein; Lp(a), lipoprotein(a); TYG, triglyceride-glucose index. HRT, hormone replacement therapy.

Table S7. Association of Reproductive Factors with Incident Ischemic Heart Disease.

Reproductive Factors	Model 1			Model 2		
	HR (95%CI)	P-value	P for trend	HR (95%CI)	P-value	P for trend
**Age at menarche, year**			<0.001			0.004
<12	1.13(1.08-1.18)	<0.001		1.06(1.01-1.11)	0.014	
12-13	Ref.			Ref.		
>13	1.08(1.02-1.15)	0.009		1.08(1.02-1.15)	0.012	
**Age at menopause[a], year**			0.064			0.104
<46	1.43(1.32-1.55)	<0.001		1.31(1.21-1.41)	<0.001	
46-49	1.14(1.06-1.23)	<0.001		1.12(1.04-1.21)	0.004	
50-51	Ref.			Ref.		
52-53	1.08(0.98-1.18)	0.108		1.07(0.98-1.18)	0.134	
>53	1.07(0.98-1.17)	0.134		1.03(0.95-1.13)	0.455	
**Reproductive lifespan[a], year**			0.207			0.158
<33	1.34(1.25-1.43)	<0.001		1.24(1.16-1.33)	<0.001	
33-35	1.10(1.02-1.19)	0.011		1.10(1.02-1.18)	0.015	
36-38	Ref.			Ref.		
39-40	1.01(0.93-1.09)	0.893		1.00(0.92-1.09)	0.957	
>40	1.05(0.97-1.13)	0.219		1.01(0.94-1.09)	0.755	
**Age at first live birth[b], year**			<0.001			<0.001
<22	1.34(1.25-1.43)	<0.001		1.20(1.12-1.28)	<0.001	
22-23	1.10(1.02-1.19)	0.011		1.06(0.98-1.15)	0.123	
24-26	Ref.			Ref.		
27-29	0.86(0.79-0.94)	<0.001		0.89(0.81-0.97)	0.008	
>29	0.74(0.66-0.82)	<0.001		0.78(0.70-0.87)	<0.001	
**Age at last live birth[b], year**			<0.001			0.046

<26	1.20(1.11-1.30)	<0.001		1.13(1.04-1.22)	0.003	
26-28	1.06(0.98-1.15)	0.165		1.04(0.96-1.12)	0.388	
29-30	Ref.			Ref.		
31-34	0.97(0.89-1.05)	0.446		0.98(0.90-1.07)	0.682	
>34	0.96(0.87-1.06)	0.423		0.99(0.90-1.09)	0.894	
**Number of live births**			<0.001			0.008
0	0.98(0.93-1.03)	0.358		0.98(0.93-1.03)	0.424	
1-2	Ref.			Ref.		
3-4	1.25(1.16-1.35)	<0.001		1.18(1.09-1.28)	<0.001	
>4	1.23(0.94-1.59)	0.126		1.11(0.85-1.44)	0.440	
**Number of stillbirths**			<0.001			0.005
0	Ref.			Ref.		
1	1.14(1.00-1.29)	0.047		1.07(0.95-1.22)	0.267	
>=2	1.74(1.31-2.32)	<0.001		1.61(1.21-2.14)	0.001	
**Number of miscarriages or terminations**			<0.001			<0.001
0	Ref.			Ref.		
1	1.07(1.02-1.13)	0.010		1.05(1.00-1.11)	0.070	
>=2	1.16(1.08-1.24)	<0.001		1.12(1.04-1.20)	0.002	
**hysterectomy**						
No	Ref.			Ref.		
Yes	1.41(1.35-1.48)	<0.001		1.26(1.20-1.32)	<0.001	
**oophorectomy**						
No	Ref.			Ref.		
Yes	1.30(1.24-1.36)	<0.001		1.10(1.05-1.16)	<0.001	

Abbreviations: HR, hazard ratio; CI, confidence interval.

Abbreviations: HR, hazard ratio; CI, confidence interval; Ref, reference.

Model 2 adjusted for covariables in model 1 plus smoking status, drinking status, body mass index, systolic blood pressure, C-reactive protein, cholesterol, high-density lipoprotein, triglyceride-glucose index, hormone replacement therapy and use of oral contraceptives.

[a]Among postmenopausal women with valid menopausal age (n = 112,203).

[b]Among parous women (more than 2 children) with live birth age information (n = 128,535).

Table S8. Association of Reproductive Factors with Incident Stroke.

Reproductive Factors	Model 1			Model 2		
	HR (95%CI)	P-value	P for trend	HR (95%CI)	P-value	P for trend
**Age at menarche, year**			<0.001			<0.001
<12	1.13(1.08-1.17)	<0.001		1.07(1.03-1.12)	<0.001	
12-13	Ref.			Ref.		
>13	1.08(1.03-1.14)	0.002		1.08(1.02-1.14)	0.005	
**Age at menopause[a], year**			0.021			0.040
<46	1.35(1.26-1.44)	<0.001		1.25(1.17-1.34)	<0.001	
46-49	1.11(1.04-1.19)	0.001		1.10(1.03-1.17)	0.006	
50-51	Ref.			Ref.		
52-53	1.03(0.96-1.12)	0.402		1.03(0.95-1.12)	0.465	
>53	1.04(0.97-1.12)	0.260		1.02(0.94-1.09)	0.682	
**Reproductive lifespan[a], year**			0.174			0.158
<33	1.30(1.22-1.38)	<0.001		1.21(1.14-1.29)	<0.001	
33-35	1.10(1.03-1.17)	0.006		1.09(1.02-1.16)	0.010	
36-38	Ref.			Ref.		
39-40	1.02(0.95-1.10)	0.521		1.02(0.95-1.10)	0.550	
>40	1.03(0.96-1.10)	0.379		1.00(0.94-1.07)	0.943	
**Age at first live birth[b], year**			<0.001			<0.001
<22	1.31(1.24-1.39)	<0.001		1.19(1.13-1.27)	<0.001	
22-23	1.08(1.01-1.16)	0.021		1.05(0.98-1.12)	0.179	
24-26	Ref.			Ref.		
27-29	0.89(0.83-0.96)	0.002		0.92(0.85-0.99)	0.021	
>29	0.78(0.71-0.86)	<0.001		0.82(0.75-0.90)	<0.001	
**Age at last live birth[b], year**			<0.001			0.049

<26	1.20(1.12-1.29)	<0.001		1.14(1.06-1.22)	<0.001	
26-28	1.08(1.01-1.16)	0.034		1.06(0.99-1.14)	0.118	
29-30	Ref.			Ref.		
31-34	0.98(0.91-1.06)	0.686		1.00(0.93-1.07)	0.945	
>34	0.97(0.89-1.06)	0.534		1.00(0.92-1.09)	0.953	
**Number of live births**			<0.001			<0.001
0	0.99(0.95-1.03)	0.596		0.99(0.95-1.03)	0.669	
1-2	Ref.			Ref.		
3-4	1.24(1.16-1.33)	<0.001		1.19(1.11-1.27)	<0.001	
>4	1.25(1.00-1.58)	0.052		1.15(0.92-1.45)	0.219	
**Number of stillbirths**			<0.001			0.002
0	Ref.			Ref.		
1	1.14(1.02-1.27)	0.021		1.08(0.97-1.21)	0.154	
>=2	1.65(1.27-2.13)	<0.001		1.54(1.19-1.99)	0.001	
**Number of miscarriages or terminations**			<0.001			<0.001
0	Ref.			Ref.		
1	1.03(0.99-1.08)	0.163		1.01(0.97-1.06)	0.537	
>=2	1.16(1.10-1.24)	<0.001		1.13(1.06-1.20)	<0.001	
**hysterectomy**						
No	Ref.			Ref.		
Yes	1.34(1.28-1.40)	<0.001		1.22(1.17-1.27)	<0.001	
**oophorectomy**						
No	Ref.			Ref.		
Yes	1.24(1.17-1.31)	<0.001		1.11(1.04-1.17)	<0.001	

Abbreviations: HR, hazard ratio; CI, confidence interval; Ref, reference.

Model 1 adjusted for age, Townsend deprived index, qualifications and ethnicity.

Model 2 adjusted for covariables in model 1 plus smoking status, drinking status, body mass index, systolic blood pressure, C-reactive protein, cholesterol, high-density lipoprotein, triglyceride-glucose index, hormone replacement therapy and use of oral contraceptives.

[a]Among postmenopausal women with valid menopausal age (n = 112,203).

[b]Among parous women (more than 2 children) with live birth age information (n = 128,535).

Table S9. Association of Reproductive Factors with Incident Type 2 Diabetes Mellitus.

Reproductive Factors	Model 1			Model 2		
	HR (95%CI)	P-value	P for trend	HR (95%CI)	P-value	P for trend
**Age at menarche, year**			<0.001			0.035
<12	1.24(1.18-1.31)	<0.001		1.01(0.96-1.07)	0.703	
12-13	Ref.			Ref.		
>13	1.05(0.97-1.12)	0.217		1.09(1.02-1.17)	0.017	
**Age at menopause[a], year**			0.341			0.137
<46	1.28(1.17-1.41)	<0.001		1.14(1.03-1.25)	0.009	
46-49	1.04(0.95-1.14)	0.380		1.04(0.95-1.13)	0.457	
50-51	Ref.			Ref.		
52-53	0.99(0.88-1.11)	0.836		0.97(0.87-1.09)	0.594	
>53	1.09(0.98-1.21)	0.122		0.99(0.89-1.10)	0.877	
**Reproductive lifespan[a], year**			0.798			0.071
<33	1.23(1.13-1.34)	<0.001		1.11(1.02-1.21)	0.018	
33-35	1.04(0.95-1.14)	0.424		1.06(0.97-1.16)	0.201	
36-38	Ref.			Ref.		
39-40	1.04(0.94-1.14)	0.486		0.99(0.89-1.09)	0.770	
>40	1.08(0.98-1.19)	0.107		0.94(0.86-1.03)	0.210	
**Age at first live birth[b], year**			<0.001			0.008
<22	1.41(1.30-1.53)	<0.001		1.15(1.06-1.24)	<0.001	
22-23	1.15(1.05-1.27)	0.002		1.07(0.98-1.18)	0.137	
24-26	Ref.			Ref.		
27-29	0.87(0.78-0.96)	0.008		0.95(0.86-1.06)	0.359	
>29	0.75(0.66-0.85)	<0.001		0.88(0.78-0.99)	0.041	
**Age at last live birth[b], year**			0.008			0.738

<26	1.22(1.11-1.34)	<0.001		1.09(0.99-1.20)	0.065	
26-28	1.04(0.94-1.14)	0.481		1.01(0.91-1.11)	0.914	
29-30	Ref.			Ref.		
31-34	0.96(0.87-1.07)	0.476		1.01(0.91-1.12)	0.823	
>34	1.01(0.91-1.13)	0.822		1.10(0.98-1.22)	0.103	
**Number of live births**			<0.001			<0.001
0	1.03(0.97-1.09)	0.344		1.02(0.96-1.08)	0.528	
1-2	Ref.			Ref.		
3-4	1.47(1.34-1.60)	<0.001		1.27(1.16-1.38)	<0.001	
>4	1.73(1.34-2.23)	<0.001		1.34(1.04-1.72)	0.023	
**Number of stillbirths**			<0.001			0.016
0	Ref.			Ref.		
1	1.30(1.13-1.49)	<0.001		1.17(1.02-1.34)	0.029	
>=2	1.37(0.95-1.98)	0.088		1.23(0.86-1.77)	0.263	
**Number of miscarriages or terminations**			<0.001			<0.001
0	Ref.			Ref.		
1	1.04(0.97-1.10)	0.259		1.03(0.97-1.09)	0.408	
>=2	1.24(1.15-1.34)	<0.001		1.20(1.12-1.30)	<0.001	
**hysterectomy**						
No	Ref.			Ref.		
Yes	1.55(1.47-1.64)	<0.001		1.22(1.15-1.29)	<0.001	
**oophorectomy**						
No	Ref.			Ref.		
Yes	1.44(1.34-1.55)	<0.001		1.12(1.04-1.21)	0.004	

Abbreviations: HR, hazard ratio; CI, confidence interval; Ref, reference.

Model 1 adjusted for age, Townsend deprived index, qualifications and ethnicity.

Model 2 adjusted for covariables in model 1 plus smoking status, drinking status, body mass index, systolic blood pressure, C-reactive protein, cholesterol, high-density lipoprotein, triglyceride-glucose index, hormone replacement therapy and use of oral contraceptives.

[a]Among postmenopausal women with valid menopausal age (n = 112,203).

[b]Among parous women (more than 2 children) with live birth age information (n = 128,535).

Table S10. Association of Reproductive Factors with Incident Cardiometabolic Disease Stratified by Age.

Reproductive Factors	Age < 65y		Age ≥ 65y		P for interaction
	HR (95%CI)	P-value	HR (95%CI)	P-value	
**Age at menarche, year**					0.014
<12	1.07 (1.03, 1.12)	<0.001	0.99 (0.93, 1.05)	0.736	
12-13	Ref.		Ref.		
>13	1.09 (1.04, 1.15)	0.001	1.05 (0.97, 1.13)	0.237	
**Age at menopause[a], year**					0.346
<46	1.14 (1.06, 1.22)	<0.001	1.19 (1.08, 1.31)	<0.001	
46-49	1.03 (0.97, 1.10)	0.358	1.13 (1.03, 1.24)	0.011	
50-51	Ref.		Ref.		
52-53	1.02 (0.94, 1.11)	0.595	1.04 (0.93, 1.17)	0.479	
>53	1.08 (1.00, 1.17)	0.052	1.08 (0.98, 1.20)	0.133	
**Reproductive lifespan[a], year**					0.337
<33	1.11 (1.04, 1.19)	0.001	1.16 (1.06, 1.26)	0.001	
33-35	1.03 (0.96, 1.10)	0.371	1.11 (1.01, 1.21)	0.030	
36-38	Ref.		Ref.		
39-40	1.02 (0.95, 1.10)	0.513	1.06 (0.96, 1.17)	0.253	
>40	1.06 (0.99, 1.14)	0.099	1.01 (0.92, 1.11)	0.816	
**Age at first live birth[b], year**					<0.001
<22	1.20 (1.13, 1.27)	0.000	1.17 (1.08, 1.27)	<0.001	
22-23	1.10 (1.02, 1.17)	0.011	1.01 (0.92, 1.11)	0.788	
24-26	Ref.		Ref.		
27-29	0.91 (0.85, 0.98)	0.016	0.98 (0.88, 1.10)	0.763	
>29	0.79 (0.73, 0.87)	0.000	0.93 (0.81, 1.07)	0.312	
**Age at last live birth[b], year**					0.006

<26	1.16 (1.08, 1.24)	0.000	1.08 (0.98, 1.19)	0.126	
26-28	1.09 (1.01, 1.17)	0.022	0.98 (0.89, 1.08)	0.627	
29-30	Ref.		Ref.		
31-34	1.00 (0.93, 1.08)	0.927	1.01 (0.90, 1.12)	0.923	
>34	1.04 (0.96, 1.13)	0.352	1.00 (0.88, 1.13)	0.989	
**Number of live births**					0.002
0	0.93 (0.89, 0.97)	0.001	1.03 (0.96, 1.10)	0.386	
1-2	Ref.		Ref.		
3-4	1.26 (1.17, 1.35)	0.000	1.20 (1.10, 1.31)	<0.001	
>4	1.40 (1.13, 1.74)	0.002	1.15 (0.86, 1.54)	0.340	
**Number of stillbirths**					0.493
0	Ref.		Ref.		
1	1.12 (0.99, 1.25)	0.064	1.05 (0.91, 1.22)	0.513	
>=2	1.33 (1.01, 1.75)	0.041	1.62 (1.13, 2.32)	0.009	
**Number of miscarriages or terminations**					0.612
0	Ref.		Ref.		
1	0.99 (0.95, 1.03)	0.624	1.02 (0.95, 1.10)	0.578	
>=2	1.10 (1.04, 1.17)	0.001	1.10 (1.00, 1.21)	0.055	
**hysterectomy**					<0.001
No	Ref.		Ref.		
Yes	1.23 (1.18, 1.29)	0.000	1.13 (1.06, 1.20)	<0.001	
**oophorectomy**					0.002
No	Ref.		Ref.		
Yes	1.11 (1.05, 1.18)	0.010	1.08 (0.99, 1.17)	0.074	

Abbreviations: HR, hazard ratio; CI, confidence interval; Ref, reference.

Model adjusted for Townsend deprived index, qualifications, ethnicity, smoking status, drinking status, body mass index, systolic blood pressure, C-reactive protein, cholesterol, high-density lipoprotein, triglyceride-glucose index, hormone replacement therapy and use of oral contraceptives.

[a]Among postmenopausal women with valid menopausal age (n = 112,203).

[b]Among parous women (more than 2 children) with live birth age information (n = 128,535).

Table S11. Association of Reproductive Factors with Incident Cardiometabolic Disease Stratified by BMI.

Reproductive Factors	Normal		Overweight[c]		Obese		P for interaction
	HR (95%CI)	P-value	HR (95%CI)	P-value	HR (95%CI)	P-value	
**Age at menarche, year**							0.259
<12	1.10 (1.02, 1.17)	0.008	1.07 (1.02, 1.13)	0.011	1.02 (0.96, 1.07)	0.528	
12-13	Ref.		Ref.		Ref.		
>13	1.06 (0.98, 1.14)	0.161	1.08 (1.00, 1.15)	0.039	1.10 (1.02, 1.19)	0.014	
**Age at menopause[a], year**							0.621
<46	1.25 (1.12, 1.40)	0.000	1.18 (1.08, 1.29)	0.000	1.22 (1.11, 1.35)	0.000	
46-49	1.09 (0.99, 1.21)	0.084	1.04 (0.95, 1.13)	0.407	1.12 (1.02, 1.23)	0.014	
50-51	Ref.		Ref.		Ref.		
52-53	1.10 (0.97, 1.25)	0.130	0.96 (0.87, 1.07)	0.507	0.98 (0.87, 1.10)	0.766	
>53	0.99 (0.87, 1.12)	0.844	1.01 (0.91, 1.11)	0.908	1.06 (0.95, 1.18)	0.288	
**Reproductive lifespan[a], year**							0.903
<33	1.22 (1.10, 1.34)	0.000	1.18 (1.08, 1.28)	0.000	1.17 (1.07, 1.27)	0.001	
33-35	1.10 (1.00, 1.22)	0.051	1.05 (0.96, 1.15)	0.269	1.10 (1.00, 1.21)	0.058	
36-38	Ref.		Ref.		Ref.		
39-40	1.07 (0.95, 1.19)	0.260	1.03 (0.93, 1.13)	0.597	0.96 (0.87, 1.06)	0.443	
>40	0.99 (0.88, 1.11)	0.808	0.99 (0.90, 1.08)	0.793	1.00 (0.91, 1.10)	0.960	
**Age at first live birth[b], year**							0.864
<22	1.11 (1.01, 1.23)	0.032	1.22 (1.13, 1.32)	0.000	1.20 (1.11, 1.30)	0.000	
22-23	0.97 (0.87, 1.09)	0.619	1.08 (0.99, 1.18)	0.082	1.11 (1.01, 1.22)	0.034	
24-26	Ref.		Ref.		Ref.		
27-29	0.95 (0.85, 1.06)	0.355	0.96 (0.87, 1.06)	0.409	0.93 (0.83, 1.03)	0.179	

>29	0.84 (0.74, 0.97)	0.014	0.92 (0.81, 1.03)	0.160	0.82 (0.71, 0.93)	0.003	
**Age at last live birth[b], year**							0.025
<26	1.07 (0.96, 1.20)	0.229	1.17 (1.07, 1.29)	0.001	1.10 (1.00, 1.21)	0.054	
26-28	0.99 (0.88, 1.11)	0.879	1.06 (0.97, 1.17)	0.195	1.05 (0.95, 1.16)	0.341	
29-30	Ref.		Ref.		Ref.		
31-34	0.97 (0.87, 1.09)	0.642	1.02 (0.93, 1.13)	0.632	1.02 (0.92, 1.13)	0.774	
>34	0.91 (0.79, 1.04)	0.157	1.13 (1.01, 1.26)	0.034	1.07 (0.95, 1.20)	0.269	
**Number of live births**							0.678
0	1.02 (0.95, 1.09)	0.616	1.03 (0.97, 1.09)	0.306	1.00 (0.94, 1.06)	0.931	
1-2	Ref.		Ref.		Ref.		
3-4	1.10 (0.97, 1.25)	0.146	1.25 (1.14, 1.37)	0.000	1.26 (1.16, 1.37)	0.000	
>4	1.17 (0.73, 1.89)	0.517	1.32 (0.98, 1.77)	0.065	1.34 (1.05, 1.70)	0.018	
**Number of stillbirths**							0.968
0	Ref.		Ref.		Ref.		
1	1.05 (0.87, 1.28)	0.594	1.02 (0.88, 1.18)	0.762	1.10 (0.95, 1.27)	0.210	
>=2	1.11 (0.62, 2.01)	0.725	1.38 (0.96, 1.97)	0.081	1.44 (1.06, 1.97)	0.021	
**Number of miscarriages or terminations**							<0.001
0	Ref.		Ref.		Ref.		
1	0.93 (0.86, 1.01)	0.083	1.06 (1.00, 1.13)	0.065	1.03 (0.96, 1.09)	0.433	
>=2	1.01 (0.91, 1.12)	0.832	1.14 (1.05, 1.24)	0.001	1.22 (1.13, 1.32)	0.000	
**hysterectomy**							<0.001
No	Ref.		Ref.		Ref.		
Yes	1.24 (1.16, 1.34)	0.000	1.25 (1.18, 1.33)	0.000	1.13 (1.07, 1.20)	0.000	
**oophorectomy**							0.015

No	Ref.		Ref.		Ref.	
Yes	1.17 (1.06, 1.29)	0.002	1.12 (1.04, 1.22)	0.004	1.13 (1.06, 1.19)	0.000

Abbreviations: HR, hazard ratio; CI, confidence interval; Ref, reference.

Model adjusted for age, Townsend deprived index, qualifications, ethnicity, smoking status, drinking status, body mass index, systolic blood pressure, C-reactive protein, cholesterol, high-density lipoprotein, triglyceride-glucose index, hormone replacement therapy and use of oral contraceptives.

[a]Among postmenopausal women with valid menopausal age (n = 112,203).

[b]Among parous women (more than 2 children) with live birth age information (n = 128,535).

Table S12. Association of Reproductive Factors with Incident Cardiometabolic Disease Stratified by Waist Circumference.

Reproductive Factors	Normal		Abdominal Obesity[c]		P for interaction
	HR (95%CI)	P-value	HR (95%CI)	P-value	
**Age at menarche, year**					0.343
<12	1.07 (1.02, 1.13)	0.005	1.02 (0.98, 1.07)	0.327	
12-13	Ref.		Ref.		
>13	1.05 (0.99, 1.12)	0.095	1.11 (1.04, 1.17)	0.001	
**Age at menopause[a], year**					0.297
<46	1.22 (1.12, 1.33)	0.000	1.21 (1.12, 1.31)	0.000	
46-49	1.07 (0.99, 1.16)	0.079	1.09 (1.01, 1.18)	0.023	
50-51	Ref.		Ref.		
52-53	1.07 (0.97, 1.18)	0.167	0.95 (0.86, 1.04)	0.284	
>53	1.00 (0.91, 1.10)	0.992	1.03 (0.94, 1.12)	0.559	
**Reproductive lifespan[a], year**					0.935
<33	1.19 (1.10, 1.28)	0.000	1.19 (1.11, 1.28)	0.000	
33-35	1.08 (1.00, 1.17)	0.044	1.08 (1.00, 1.17)	0.044	
36-38	Ref.		Ref.		
39-40	1.00 (0.92, 1.09)	0.916	1.02 (0.94, 1.10)	0.689	
>40	1.00 (0.92, 1.09)	0.920	0.97 (0.90, 1.05)	0.459	
**Age at first live birth[b], year**					0.784
<22	1.17 (1.09, 1.26)	0.000	1.19 (1.12, 1.27)	0.000	
22-23	1.03 (0.95, 1.12)	0.467	1.09 (1.01, 1.18)	0.030	
24-26	Ref.		Ref.		
27-29	0.95 (0.87, 1.03)	0.215	0.94 (0.86, 1.02)	0.144	

>29	0.84 (0.75, 0.93)	0.001	0.87 (0.78, 0.96)	0.008	
**Age at last live birth[b], year**					0.243
<26	1.13 (1.03, 1.23)	0.006	1.11 (1.03, 1.20)	0.007	
26-28	1.03 (0.95, 1.12)	0.472	1.05 (0.97, 1.14)	0.204	
29-30	Ref.		Ref.		
31-34	1.00 (0.92, 1.10)	0.923	1.01 (0.92, 1.09)	0.894	
>34	0.99 (0.89, 1.10)	0.822	1.08 (0.98, 1.18)	0.116	
**Number of live births**					0.873
0	1.02 (0.97, 1.08)	0.367	0.99 (0.95, 1.04)	0.800	
1-2	Ref.		Ref.		
3-4	1.17 (1.06, 1.28)	0.001	1.24 (1.16, 1.33)	0.000	
>4	1.33 (0.97, 1.82)	0.081	1.26 (1.02, 1.55)	0.031	
**Number of stillbirths**					0.977
0	Ref.		Ref.		
1	1.04 (0.91, 1.20)	0.574	1.08 (0.95, 1.21)	0.230	
>=2	1.33 (0.92, 1.93)	0.131	1.43 (1.09, 1.87)	0.009	
**Number of miscarriages or terminations**					<0.001
0	Ref.		Ref.		
1	0.99 (0.93, 1.05)	0.713	1.03 (0.98, 1.09)	0.232	
>=2	1.03 (0.95, 1.11)	0.476	1.23 (1.15, 1.31)	0.000	
**hysterectomy**					0.007
No	Ref.		Ref.		
Yes	1.24 (1.18, 1.31)	0.000	1.17 (1.12, 1.23)	0.000	
**oophorectomy**					0.204

No	Ref.		Ref.	
Yes	1.12 (1.04, 1.21)	0.002	1.09 (1.02, 1.16)	0.007

Abbreviations: HR, hazard ratio; CI, confidence interval; Ref, reference.

Model adjusted for age, Townsend deprived index, qualifications, ethnicity, smoking status, drinking status, body mass index, systolic blood pressure, C-reactive protein, cholesterol, high-density lipoprotein, triglyceride-glucose index, hormone replacement therapy and use of oral contraceptives.

[a]Among postmenopausal women with valid menopausal age (n = 112,203).

[b]Among parous women (more than 2 children) with live birth age information (n = 128,535).

Table S13. Association of Reproductive Factors with Incident Cardiometabolic Disease Stratified by Smoking Status.

Reproductive Factors	Non-Smoking		Smokers		P for interaction
	HR (95%CI)	P-value	HR (95%CI)	P-value	
**Age at menarche, year**					0.090
<12	1.01 (0.96, 1.05)	0.756	1.09 (1.04, 1.14)	0.001	
12-13	Ref.		Ref.		
>13	1.10 (1.03, 1.16)	0.003	1.07 (1.00, 1.14)	0.042	
**Age at menopause[a], year**					0.104
<46	1.20 (1.11, 1.30)	<0.001	1.23 (1.14, 1.34)	<0.001	
46-49	1.06 (0.99, 1.14)	0.115	1.11 (1.02, 1.20)	0.011	
50-51	Ref.		Ref.		
52-53	0.97 (0.89, 1.06)	0.515	1.05 (0.95, 1.17)	0.309	
>53	1.04 (0.95, 1.12)	0.411	0.99 (0.90, 1.09)	0.802	
**Reproductive lifespan[a], year**					0.655
<33	1.17 (1.09, 1.26)	<0.001	1.20 (1.11, 1.29)	<0.001	
33-35	1.08 (1.00, 1.16)	0.046	1.09 (1.01, 1.18)	0.036	
36-38	Ref.		Ref.		
39-40	1.02 (0.95, 1.11)	0.551	0.99 (0.91, 1.09)	0.885	
>40	0.97 (0.90, 1.05)	0.507	1.00 (0.92, 1.09)	0.954	
**Age at first live birth[b], year**					0.491
<22	1.17 (1.10, 1.25)	<0.001	1.19 (1.11, 1.28)	<0.001	
22-23	1.04 (0.97, 1.12)	0.251	1.08 (0.99, 1.18)	0.072	
24-26	Ref.		Ref.		
27-29	0.95 (0.87, 1.03)	0.184	0.93 (0.85, 1.03)	0.162	
>29	0.83 (0.75, 0.92)	<0.001	0.88 (0.79, 0.99)	0.037	
**Age at last live birth[b], year**					0.166

<26	1.08 (1.00, 1.17)	0.058	1.17 (1.07, 1.27)	<0.001	
26-28	1.00 (0.93, 1.08)	0.938	1.10 (1.01, 1.20)	0.036	
29-30	Ref.		Ref.		
31-34	1.00 (0.93, 1.09)	0.941	1.01 (0.92, 1.11)	0.795	
>34	1.01 (0.92, 1.11)	0.850	1.08 (0.98, 1.20)	0.133	
**Number of live births**					0.672
0	1.02 (0.97, 1.07)	0.391	1.01 (0.96, 1.06)	0.819	
1-2	Ref.		Ref.		
3-4	1.27 (1.17, 1.37)	<0.001	1.16 (1.07, 1.25)	<0.001	
>4	1.30 (1.03, 1.65)	0.030	1.24 (0.96, 1.60)	0.100	
**Number of stillbirths**					0.080
0	Ref.		Ref.		
1	1.14 (1.01, 1.29)	0.041	0.98 (0.86, 1.12)	0.785	
>=2	1.13 (0.80, 1.61)	0.476	1.61 (1.21, 2.13)	0.001	
**Number of miscarriages or terminations**					0.145
0	Ref.		Ref.		
1	1.05 (0.99, 1.10)	0.097	0.98 (0.93, 1.03)	0.447	
>=2	1.12 (1.04, 1.20)	0.002	1.16 (1.09, 1.24)	0.000	
**hysterectomy**					0.124
No	Ref.		Ref.		
Yes	1.16 (1.11, 1.22)	<0.001	1.24 (1.17, 1.30)	<0.001	
**oophorectomy**					0.639
No	Ref.		Ref.		
Yes	1.09 (1.02, 1.16)	0.014	1.12 (1.05, 1.20)	0.001	

Abbreviations: HR, hazard ratio; CI, confidence interval; Ref, reference.

Model adjusted for age, Townsend deprived index, qualifications, ethnicity, drinking status, body mass index, systolic blood pressure, C-reactive protein, cholesterol, high-density lipoprotein, triglyceride-glucose index, hormone replacement therapy and use of oral contraceptives.

[a]Among postmenopausal women with valid menopausal age (n = 112,203).

[b]Among parous women (more than 2 children) with live birth age information (n = 128,535).

Table S14. Association of Menarche Age with Incident Cardiometabolic Disease Further adjusted by Reproductive Lifespan.

**Reproductive Factors**	**HR (95%CI)**	**P-value**
**Age at menarche, year**		
<12	1.06(1.02-1.10)	0.004
12-13	Ref.	
>13	1.00(0.95-1.06)	0.900

Abbreviations: HR, hazard ratio; CI, confidence interval; Ref, reference.

Model adjusted for age, Townsend deprived index, qualifications, ethnicity, smoking status, drinking status, body mass index, systolic blood pressure, C-reactive protein, cholesterol, high-density lipoprotein, triglyceride-glucose index, hormone replacement therapy, use of oral contraceptives and reproductive lifespan.

Table S15. Association of Reproductive Lifespan with Incident Cardiometabolic Disease Further adjusted by Menarche Age.

Reproductive Factors	HR (95%CI)	P-value
**Reproductive lifespan, year**		
<33	1.20(1.14-1.26)	<0.001
33-35	1.09(1.03-1.15)	0.001
36-38	Ref.	
39-40	1.01(0.95-1.07)	0.792
>40	0.97(0.92-1.03)	0.359

Abbreviations: HR, hazard ratio; CI, confidence interval; Ref, reference.

Model adjusted for age, Townsend deprived index, qualifications, ethnicity, smoking status, drinking status, body mass index, systolic blood pressure, C-reactive protein, cholesterol, high-density lipoprotein, triglyceride-glucose index, hormone replacement therapy, use of oral contraceptives and menarche age.

Table S16. Association of Reproductive Factors with Incident Cardiometabolic Disease Further adjusted Live Births.

Reproductive Factors	HR (95%CI)	P-value
**Age at menarche, year**		
<12	1.05(1.01-1.08)	0.008
12-13	Ref.	
>13	1.08(1.03-1.13)	<0.001
**Age at menopause[a], year**		
<46	1.22(1.15-1.29)	<0.001
46-49	1.08(1.03-1.14)	0.003
50-51	Ref.	
52-53	1.01(0.94-1.08)	0.877
>53	1.01(0.95-1.08)	0.678
**Reproductive lifespan[a], year**		
<33	1.19(1.13-1.25)	<0.001
33-35	1.08(1.03-1.14)	0.004
36-38	Ref.	
39-40	1.01(0.95-1.07)	0.709
>40	0.98(0.93-1.04)	0.543
**Age at first live birth[b], year**		
<22	1.15(1.10-1.21)	<0.001
22-23	1.05(1.00-1.11)	0.065
24-26	Ref.	
27-29	0.95(0.89-1.01)	0.075
>29	0.86(0.80-0.93)	<0.001
**Age at last live birth[b], year**		
<26	1.15(1.09-1.22)	<0.001

26-28	1.06(1.00-1.12)	0.071
29-30	Ref.	
31-34	0.99(0.93-1.05)	0.761
>34	1.00(0.93-1.07)	0.900
**Number of stillbirths**		
0	Ref.	
1	1.04(0.95-1.14)	0.360
>=2	1.37(1.10-1.70)	0.005
**Number of miscarriages or terminations**		
0	Ref.	
1	1.00(0.97-1.04)	0.851
>=2	1.13(1.07-1.19)	<0.001
**hysterectomy**		
No	Ref.	
Yes	1.19(1.15-1.24)	<0.001
**oophorectomy**		
No	Ref.	
Yes	1.10(1.05-1.16)	<0.001

Abbreviations: HR, hazard ratio; CI, confidence interval; Ref, reference.

Model adjusted for age, Townsend deprived index, qualifications, ethnicity, smoking status, drinking status, body mass index, systolic blood pressure, C-reactive protein, cholesterol, high-density lipoprotein, triglyceride-glucose index, hormone replacement therapy, use of oral contraceptives and number of live births.

[a]Among postmenopausal women with valid menopausal age (n = 112,203).

[b]Among parous women (more than 2 children) with live birth age information (n = 128,535).

Table S17. Associations between reproductive lifespan and incident cardiometabolic disease using menopause age redefined to include surgical menopause and HRT.

Reproductive Factors	Model 1		Model 2	
	**HR (95%CI)**	**P-value**	**HR (95%CI)**	**P-value**
**Reproductive lifespan, year**				
<35	1.41 (1.30-1.52)	<0.001	1.30 (1.20-1.40)	<0.001
35-38	1.14 (1.05-1.23)	0.002	1.11 (1.03-1.21)	0.008
39-41	Ref.		Ref.	
42-45	1.02 (0.95-1.11)	0.535	1.03 (0.95-1.11)	0.464
>45	1.10 (1.02-1.20)	0.017	1.10 (1.02-1.20)	0.017

Abbreviations: HR, hazard ratio; CI, confidence interval; Ref, reference.

Model 1 adjusted for age, Townsend deprived index, qualifications and ethnicity.

Model 2 adjusted for covariables in model 1 plus smoking status, drinking status, body mass index, systolic blood pressure, C-reactive protein, Total cholesterol, high-density lipoprotein, triglyceride-glucose index.

Table S18. Association of Age at First Live Birth with Incident Cardiometabolic Disease When Considering All Parous Women.

Reproductive Factors	HR (95%CI)	P-value
**Age at first live birth[a], year**		
<21	1.22 (1.16, 1.29)	<0.001
21-23	1.09 (1.04, 1.15)	<0.001
24-25	Ref.	
26-28	0.98 (0.92, 1.03)	0.417
>28	0.90 (0.85, 0.96)	<0.001

Abbreviations: HR, hazard ratio; CI, confidence interval; Ref, reference.

Model adjusted for age, Townsend deprived index, qualifications, ethnicity, smoking status, drinking status, body mass index, systolic blood pressure, C-reactive protein, cholesterol, high-density lipoprotein, triglyceride-glucose index, hormone replacement therapy and use of oral contraceptives.

[a]Among all parous women with live birth age information (n = 153,690)

Table S19. Associations of distinct pregnancy-loss categories with incident cardiometabolic disease.

Pregnancy-loss Categories	HR (95%CI)	P-value
**Number of miscarriages**		
0	Ref.	
1	1.05 (1.00-1.09)	0.037
>=2	1.22 (1.15-1.30)	<0.001
**Number of terminations**		
0	Ref.	
1	0.97 (0.92-1.02)	0.216
>=2	1.14 (1.04-1.26)	0.006

Abbreviations: HR, hazard ratio; CI, confidence interval; Ref, reference.

Model adjusted for age, Townsend deprived index, qualifications, ethnicity smoking status, drinking status, body mass index, systolic blood pressure, cholesterol, high-density lipoprotein, hormone replacement therapy and use of oral contraceptives.

Table S20. Association of Reproductive Factors with Incident Cardiometabolic Disease after excluding CRP and TyG from the adjustment set

Reproductive Factors	HR (95%CI)	P-value
**Age at menarche, year**		
<12	1.04 (1.01, 1.08)	0.011
12-13	Ref.	
>13	1.08 (1.04, 1.13)	<0.001
**Age at menopause[a], year**		
<46	1.23 (1.16, 1.30)	<0.001
46-49	1.08 (1.03, 1.14)	0.003
50-51	Ref.	
52-53	1.01 (0.94, 1.08)	0.835
>53	1.02 (0.96, 1.08)	0.571
**Reproductive lifespan[a], year**		
<33	1.20 (1.14, 1.26)	<0.001
33-35	1.09 (1.03, 1.15)	0.003
36-38	Ref.	
39-40	1.01 (0.96, 1.07)	0.665
>40	0.99 (0.93, 1.04)	0.620
**Age at first live birth[b], year**		
<22	1.19 (1.13, 1.24)	<0.001
22-23	1.06 (1.01, 1.13)	0.029
24-26	Ref.	
27-29	0.94 (0.88, 1.00)	0.037
>29	0.85 (0.79, 0.91)	<0.001
**Age at last live birth[b], year**		

<26	1.12 (1.06, 1.19)	<0.001
26-28	1.04 (0.99, 1.11)	0.142
29-30	Ref.	
31-34	1.00 (0.94, 1.07)	0.951
>34	1.03 (0.96, 1.10)	0.397
**Number of live births**		
0	1.01 (0.98, 1.05)	0.421
1-2	Ref.	
3-4	1.21 (1.14, 1.28)	<0.001
>4	1.25 (1.05, 1.49)	0.011
**Number of stillbirths**		
0	Ref.	
1	1.07 (0.98, 1.17)	0.156
>=2	1.38 (1.11, 1.71)	0.004
**Number of miscarriages or terminations**		
0	Ref.	
1	1.01 (0.97, 1.05)	0.607
>=2	1.14 (1.09, 1.20)	<0.001
**hysterectomy**		
No	Ref.	
Yes	1.23 (1.19, 1.28)	<0.001
**oophorectomy**		
No	Ref.	
Yes	1.14 (1.08, 1.19)	<0.001

Abbreviations: HR, hazard ratio; CI, confidence interval; Ref, reference.

Adjusted for age, Townsend deprived index, qualifications, ethnicity smoking status, drinking status, body mass index, systolic blood pressure,

cholesterol, high-density lipoprotein, hormone replacement therapy and use of oral contraceptives.

[a]Among postmenopausal women with valid menopausal age (n = 112,203).

[b]Among parous women (more than 2 children) with live birth age information (n = 128,535).

Figure S1. Flowchart of the study population.

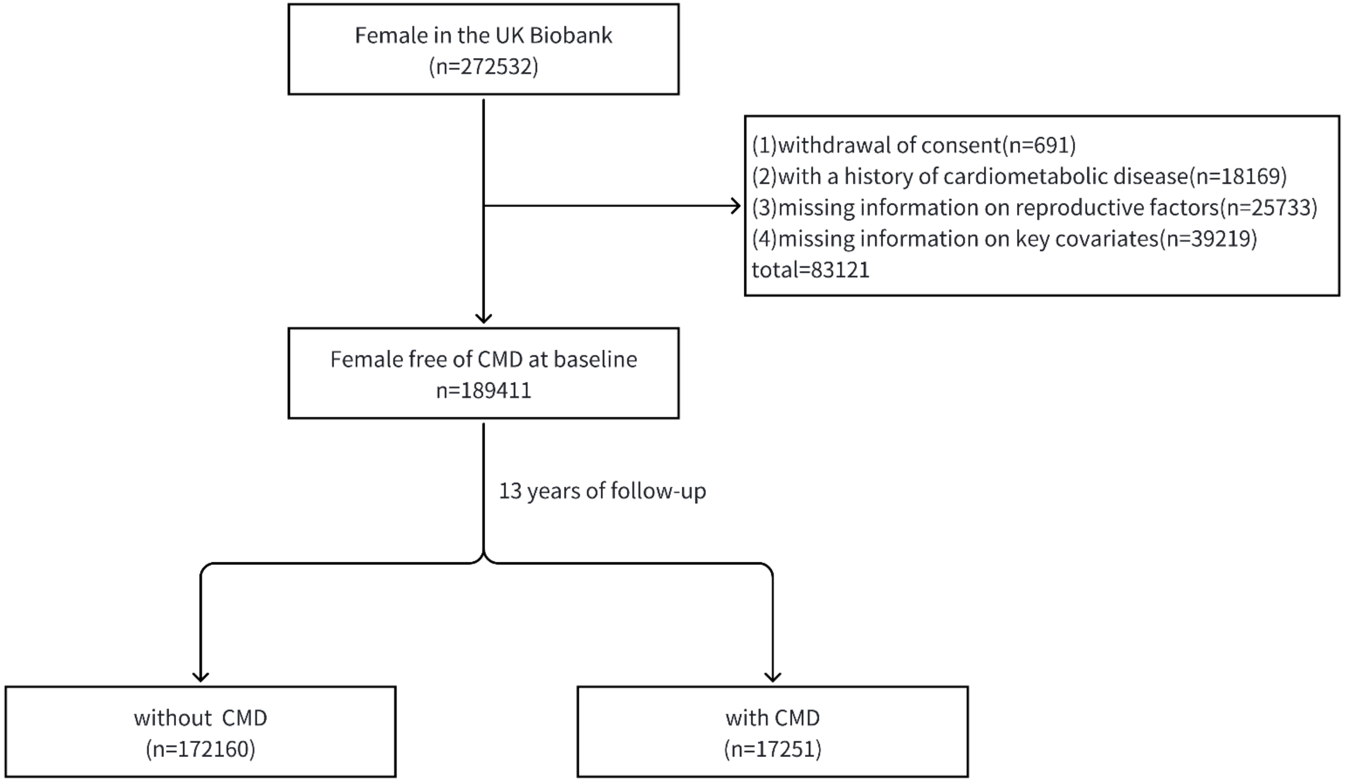

Abbreviations: CMD, cardiometabolic disease.

Figure S2. Causal directed acyclic graph of the association between reproductive factors and cardiometabolic disease.

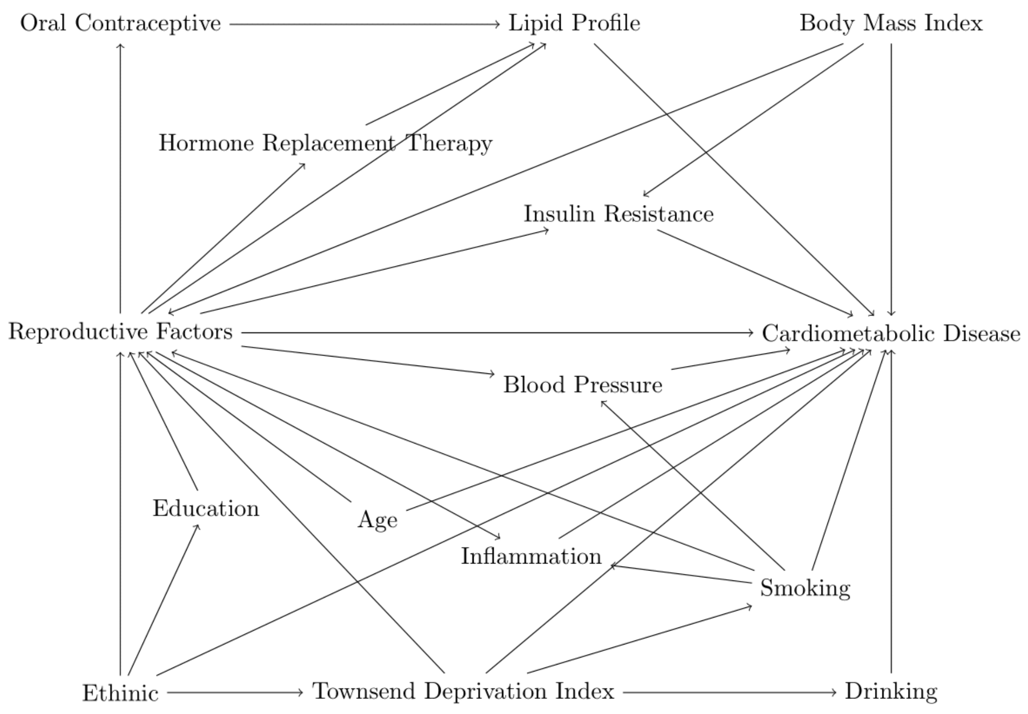

Note: The minimal sufficient adjustment set includes age, Townsend deprivation index, ethnicity, education, smoking status, alcohol consumption, BMI, inflammation level, lipid profile and insulin resistance

Figure S3. Forest plot of hazard ratios and 95% CIs for the association of reproductive factors and cardiometabolic disease stratified by waist circumference.

Reproductive Factors	Event/N	Normal	HR(95%CI)	Event/N	Abdominal obesity	HR(95%CI)	P interaction
Age at menarche, year							0.343
<12	2944/44412		1.07 (1.02, 1.13)	4201/28797		1.02 (0.98, 1.07)	
12-13	3426/57434		1.00 (1.00, 1.00)	3702/27118		1.00 (1.00, 1.00)	
>13	1448/21755		1.05 (0.99, 1.12)	1528/9864		1.11 (1.04, 1.17)	
Age at menopause, year							0.297
<46	1323/14328		1.22 (1.12, 1.33)	1589/8739		1.21 (1.12, 1.31)	
46-49	1759/23193		1.07 (0.99, 1.16)	1898/12176		1.09 (1.01, 1.18)	
50-51	1018/14639		1.00 (1.00, 1.00)	1068/7446		1.00 (1.00, 1.00)	
52-53	724/9541		1.07 (0.97, 1.18)	716/5227		0.95 (0.86, 1.04)	
>53	859/10206		1.00 (0.91, 1.10)	1086/6695		1.03 (0.94, 1.12)	
Reproductive lifespan, year							0.935
<33	1362/15001		1.19 (1.10, 1.28)	1572/8717		1.19 (1.11, 1.28)	
33-35	1119/14432		1.08 (1.00, 1.17)	1141/7279		1.08 (1.00, 1.17)	
36-38	1519/21022		1.00 (1.00, 1.00)	1573/10767		1.00 (1.00, 1.00)	
39-40	798/10713		1.00 (0.92, 1.09)	918/6099		1.02 (0.94, 1.10)	
>40	885/10739		1.00 (0.92, 1.09)	1153/7421		0.97 (0.90, 1.05)	
Age at first live birth, year							0.784
<22	1935/20126		1.17 (1.09, 1.26)	2859/15024		1.19 (1.12, 1.27)	
22-23	1017/12998		1.03 (0.95, 1.12)	1226/7624		1.09 (1.01, 1.18)	
24-26	1373/21282		1.00 (1.00, 1.00)	1434/10671		1.00 (1.00, 1.00)	
27-29	812/16251		0.95 (0.87, 1.03)	790/7012		0.94 (0.86, 1.02)	
>29	455/12495		0.84 (0.75, 0.93)	479/5037		0.87 (0.78, 0.96)	
Age at last live birth, year							0.243
<26	1562/17184		1.13 (1.03, 1.23)	2032/11468		1.11 (1.03, 1.20)	
26-28	1409/18901		1.03 (0.95, 1.12)	1657/10553		1.05 (0.97, 1.14)	
29-30	848/13449		1.00 (1.00, 1.00)	939/6714		1.00 (1.00, 1.00)	
31-34	1119/20214		1.00 (0.92, 1.10)	1292/9959		1.01 (0.92, 1.09)	
>34	654/13404		0.99 (0.89, 1.10)	868/6674		1.08 (0.98, 1.18)	
Number of live births							0.873
0	2226/40449		1.02 (0.97, 1.08)	2643/20411		0.99 (0.95, 1.04)	
1-2	5033/77005		1.00 (1.00, 1.00)	5778/40404		1.00 (1.00, 1.00)	
3-4	520/5805		1.17 (1.06, 1.28)	919/4557		1.24 (1.16, 1.33)	
>4	39/342		1.33 (0.97, 1.82)	91/407		1.26 (1.02, 1.55)	
Number of stillbirths							0.977
0	7586/120991		1.00 (1.00, 1.00)	9102/63997		1.00 (1.00, 1.00)	
1	204/2344		1.04 (0.91, 1.20)	276/1545		1.08 (0.95, 1.21)	
≥2	28/266		1.33 (0.92, 1.93)	53/237		1.43 (1.09, 1.87)	
Number of miscarriages or terminations							<0.001
0	5544/85370		1.00 (1.00, 1.00)	6362/44770		1.00 (1.00, 1.00)	
1	1524/25532		0.99 (0.93, 1.05)	1927/13812		1.03 (0.98, 1.09)	
≥2	750/12699		1.03 (0.95, 1.11)	1142/7197		1.23 (1.15, 1.31)	
hysterectomy							0.007
No	5903/104925		1.00 (1.00, 1.00)	6791/51936		1.00 (1.00, 1.00)	
Yes	1915/18676		1.24 (1.18, 1.31)	2640/13843		1.17 (1.12, 1.23)	
oophorectomy							0.204
No	6988/115155		1.00 (1.00, 1.00)	8232/59399		1.00 (1.00, 1.00)	
Yes	830/8446		1.12 (1.04, 1.21)	1199/6380		1.09 (1.02, 1.16)	

0.8 1 1.2 1.4 — Lower risk / Higher risk (both panels)

Abbreviations: HR, hazard ratio; CI, confidence interval.

Adjusted for age, Townsend deprived index, qualifications, ethnicity, smoking status, drinking status, body mass index, systolic blood pressure, C-reactive protein, cholesterol, high-density lipoprotein, triglyceride-glucose index, hormone replacement therapy and use of oral contraceptives.

Figure S4. Forest plot of hazard ratios and 95% CIs for the association of reproductive factors and cardiometabolic disease stratified by smoking status.

Reproductive Factors	Event/N	Non Smokers	HR(95%CI)	Event/N	Smokers	HR(95%CI)	P interaction
Age at menarche, year							0.09
<12	3820/44553		1.01 (0.96, 1.05)	3327/28669		1.09 (1.04, 1.14)	
12-13	3837/50842		1.00 (1.00, 1.00)	3291/33723		1.00 (1.00, 1.00)	
>13	1536/18172		1.10 (1.03, 1.16)	1440/13452		1.07 (1.00, 1.14)	
Age at menopause, year							0.104
<46	1375/12321		1.20 (1.11, 1.30)	1537/10749		1.23 (1.14, 1.34)	
46-49	1887/20477		1.06 (0.99, 1.14)	1771/14894		1.11 (1.02, 1.20)	
50-51	1151/13292		1.00 (1.00, 1.00)	935/8800		1.00 (1.00, 1.00)	
52-53	801/9089		0.97 (0.89, 1.06)	639/5680		1.05 (0.95, 1.17)	
>53	1121/10207		1.04 (0.95, 1.12)	824/6694		0.99 (0.90, 1.09)	
Reproductive lifespan, year							0.655
<33	1377/12674		1.17 (1.09, 1.26)	1557/11047		1.20 (1.11, 1.29)	
33-35	1155/12416		1.08 (1.00, 1.16)	1106/9296		1.09 (1.01, 1.18)	
36-38	1663/18804		1.00 (1.00, 1.00)	1429/12993		1.00 (1.00, 1.00)	
39-40	991/10398		1.02 (0.95, 1.11)	725/6414		0.99 (0.91, 1.09)	
>40	1149/11094		0.97 (0.90, 1.05)	889/7067		1.00 (0.92, 1.09)	
Age at first live birth, year							0.491
<22	2211/18062		1.17 (1.10, 1.25)	2583/17091		1.19 (1.11, 1.28)	
22-23	1256/12730		1.04 (0.97, 1.12)	987/7895		1.08 (0.99, 1.18)	
24-26	1698/20738		1.00 (1.00, 1.00)	1109/11219		1.00 (1.00, 1.00)	
27-29	963/15236		0.95 (0.87, 1.03)	639/8029		0.93 (0.85, 1.03)	
>29	528/10959		0.83 (0.75, 0.92)	406/6576		0.88 (0.79, 0.99)	
Age at last live birth, year							0.166
<26	1701/15527		1.08 (1.00, 1.17)	1893/13127		1.17 (1.07, 1.27)	
26-28	1674/18166		1.00 (0.93, 1.08)	1392/11291		1.10 (1.01, 1.20)	
29-30	1050/12841		1.00 (1.00, 1.00)	737/7325		1.00 (1.00, 1.00)	
31-34	1393/18996		1.00 (0.93, 1.09)	1018/11181		1.01 (0.92, 1.11)	
>34	838/12195		1.01 (0.92, 1.11)	684/7886		1.08 (0.98, 1.20)	
Number of live births							0.672
0	2537/35842		1.02 (0.97, 1.07)	2334/25034		1.01 (0.96, 1.06)	
1-2	5813/71320		1.00 (1.00, 1.00)	4998/46104		1.00 (1.00, 1.00)	
3-4	773/5961		1.27 (1.17, 1.37)	666/4401		1.16 (1.07, 1.25)	
>4	70/444		1.30 (1.03, 1.65)	60/305		1.24 (0.96, 1.60)	
Number of stillbirths							0.08
0	8901/111119		1.00 (1.00, 1.00)	7788/73899		1.00 (1.00, 1.00)	
1	260/2180		1.14 (1.01, 1.29)	221/1710		0.98 (0.86, 1.12)	
≥2	32/268		1.13 (0.80, 1.61)	49/235		1.61 (1.21, 2.13)	
Number of miscarriages or terminations							0.145
0	6586/81302		1.00 (1.00, 1.00)	5321/48857		1.00 (1.00, 1.00)	
1	1757/22000		1.05 (0.99, 1.10)	1695/17354		0.98 (0.93, 1.03)	
≥2	850/10265		1.12 (1.04, 1.20)	1042/9633		1.16 (1.09, 1.24)	
hysterectomy							0.124
No	6861/94899		1.00 (1.00, 1.00)	5835/61991		1.00 (1.00, 1.00)	
Yes	2332/18668		1.16 (1.11, 1.22)	2223/13853		1.24 (1.17, 1.30)	
oophorectomy							0.639
No	8159/105100		1.00 (1.00, 1.00)	7063/69484		1.00 (1.00, 1.00)	
Yes	1034/8467		1.09 (1.02, 1.16)	995/6360		1.12 (1.05, 1.20)	

Axes: 0.8 1 1.2 1.4 — ← Lower risk | Higher risk → (both panels)

Abbreviations: HR, hazard ratio; CI, confidence interval.

Adjusted for age, Townsend deprived index, qualifications, ethnicity, drinking status, body mass index, systolic blood pressure, C-reactive protein, cholesterol, high-density lipoprotein, triglyceride-glucose index, hormone replacement therapy and use of oral contraceptives.

Figure S5. Correlation Heatmap of Reproductive Factors.

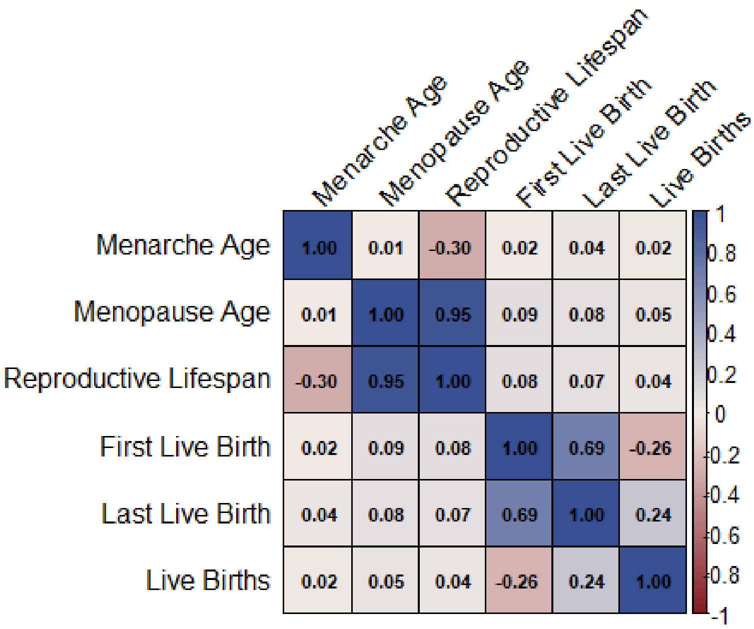

Note: This correlation matrix displays the pairwise Pearson correlation coefficients among reproductive factors. The coefficient values range from -1 to 1, with darker red indicating a strong negative correlation, darker blue indicating a strong positive correlation, and intermediate colors indicating a weaker relationship. The numbers within each cell represent the corresponding correlation coefficients.
